# Adaptation of the *Haloarcula hispanica* CRISPR-Cas system to a purified virus strictly requires a priming process

**DOI:** 10.1093/nar/gkt1154

**Published:** 2013-11-20

**Authors:** Ming Li, Rui Wang, Dahe Zhao, Hua Xiang

**Affiliations:** ^1^State Key Laboratory of Microbial Resources, Institute of Microbiology, Chinese Academy of Sciences, Beijing 100101, China and ^2^University of Chinese Academy of Sciences, Beijing 100049, China

## Abstract

The clustered regularly interspaced short palindromic repeat (CRISPR)-Cas system mediates adaptive immunity against foreign nucleic acids in prokaryotes. However, efficient adaptation of a native CRISPR to purified viruses has only been observed for the type II-A system from a *Streptococcus thermophilus* industry strain, and rarely reported for laboratory strains. Here, we provide a second native system showing efficient adaptation. Infected by a newly isolated virus HHPV-2, *Haloarcula hispanica* type I-B CRISPR system acquired spacers discriminatively from viral sequences. Unexpectedly, in addition to Cas1, Cas2 and Cas4, this process also requires Cas3 and at least partial Cascade proteins, which are involved in interference and/or CRISPR RNA maturation. Intriguingly, a preexisting spacer partially matching a viral sequence is also required, and spacer acquisition from upstream and downstream sequences of its target sequence (i.e. priming protospacer) shows different strand bias. These evidences strongly indicate that adaptation in this system strictly requires a priming process. This requirement, if validated also true for other CRISPR systems as implied by our bioinformatic analysis, may help to explain failures to observe efficient adaptation to purified viruses in many laboratory strains, and the discrimination mechanism at the adaptation level that has confused scientists for years.

## INTRODUCTION

Clustered regularly interspaced short palindromic repeats (CRISPRs), together with CRISPR-associated (Cas) proteins, comprise a recently discovered prokaryotic adaptive immune system against invasive genetic elements (1–3). Repeat sequences are intervened by spacers, which are often plasmid- or phage-derived (4–6). CRISPR RNAs (crRNAs) containing invader information are produced and used to direct the Cas machinery to interfere foreign DNA/RNA based on complementarity (7,8). Despite these common features, this system is highly diversified and has been classified into dozens of different types (9).

Extensive studies have focused on the crRNA biogenesis and interference pathways, with limited insights into how new spacers are acquired. Efficient spacer acquisition was first observed in *Streptococcus thermophilus* DGCC7710, a wildly used industry strain (10). However, studies on adaptation in laboratory strains have not been reported until 2012 (11). In the *Escherichia coli* type I-E system, overexpressed Cas1 and Cas2 have proved able to mediate inefficient naïve spacer acquisition, occasionally from the host DNA (12). This process was also observed when the expression of Cas proteins was derepressed in another two *E. coli* studies (13,14). The latter two studies also reported a priming process where a newly acquired spacer (termed priming spacer) directs subsequent spacer acquisition efficiently and specifically from the invader DNA. Different from naïve adaptation, priming adaptation also requires proteins involved in interference and exhibits an evident strand bias directed by the priming spacer (13,14). Another study on the *Sulfolobus solfataricus* CRISPR system has observed efficient adaptation to an environmental virus mixture (15). In *Pseudomonas aeruginosa*, spacer acquisition occurred inefficiently when cells were infected by an engineered lytic phage (16).

The type I-B CRISPR-Cas system in haloarchaeal cells has been shown to be highly transcribed (17) and able to provide immunity against a plasmid target (18). In this study, we provide a haloarchaeal host–virus system, which shows efficient adaptation to a purified virus without engineering CRISPR or *cas* genes. Genetic studies revealed the requirements for Cas3, Cascade protein(s), and a preexisting spacer partially matching the viral DNA. Thus, we present evidences that adaptation mediated by this native CRISPR system strictly requires a priming process.

## MATERIALS AND METHODS

### Sequences, strains and plasmids

The HHPV-2 viral genome sequence was deposited in the GenBank database under the accession number KF056323.

*Haloarcula hispanica* strains and plasmids used in this study are listed in Supplementary Table S1. The strain DF60 (Δ*pyrF* strain of *H. hispanica* ATCC 33960) and its derivative mutants were cultured at 37°C in AS-168 medium (per liter, 200 g NaCl, 20 g MgSO_4_ 7H_2_O, 2 g KCl, 3 g trisodium citrate, 1 g sodium glutamate, 50 mg FeSO_4_ 7H_2_O, 0.36 mg MnCl_2_ 4H_2_O, 5 g Bacto casamino acids, 5 g yeast extract, pH 7.2) with uracil added to a final concentration of 50 mg/liter. Strains transformed with an expression plasmid were cultured in yeast extract-subtracted AS-168.

*E. coli* JM109 was cultured in Luria–Bertani medium and used for cloning. Ampicillin was added to a final concentration of 100 mg/liter when needed.

### Halovirus isolation, purification and characterization

The water sample from a saltern on Hulu Island (Liaoning, China) was mixed with a mid-exponential *H. hispanica* culture (3:1) and screened for plaques on top agar, as previously described (19). After 6-day incubation at 30°C, a single plaque was picked and purified by several single plaque purification steps.

Top agar containing virions was inoculated into an early exponential *H. hispanica* culture for enrichment. After 6-day incubation with aeration, the culture was collected and the cells were removed by centrifugation (10 000 rpm, 4°C, 15 min). The supernatant was subjected to a 0.25 μm filter and subsequently to the VIVAFLOW 50 system (Sartorius, 50 000 MWCO) for pre-purification and concentration. Purification was performed by rate zonal centrifugation in a sucrose gradient of 10–20% (w/v), followed by a second centrifugation in a gradient of 10–50% (w/v) (SW41TI, 28 000 rpm, 8 h, 4°C). The light scattering virus band was collected and diluted with 18% (w/v) salt water. Titers were determined by plaque assays.

For transmission electron microscopy observations, the purified virions were allowed to adsorb to the grid for 1.5–2 min, and then stained with 2% (w/v) uranyl acetate for 15–30 s. Micrographs were taken with a JEM-1400 electron microscope at 120 kV in the EM unit of the Institute of Microbiology, Chinese Academy of Science.

For growth curve plotting, 1 ml of exponential *H. hispanica* culture was infected by purified HHPV-2 at a multiplicity of infection of 1. The mixture was incubated at room temperature without shaking for 5 min and then in a shaker (200 rpm, 37°C) for 25 min. Dissociative virions were removed by centrifugation, and cells were suspended and inoculated into 100 ml of fresh medium. For the uninfected control, the same treatment was performed except that 18% (w/v) salt water containing no viruses was used for mock infection. Growth curve was plotted using a DU800 spectrophotometer, with three replicates for each condition.

### Viral genome extraction, analysis and sequencing

The viral genome was isolated from the purified virions. Briefly, the virion architecture was destroyed by addition of distilled water and proteinase K, followed by extraction with phenyl–chloroform (1:1 v:v). Nucleic acids in the aqueous phase were precipitated by adding 1/10 volume of 3 M sodium acetate (pH 4.8) and 2 volumes of ethanol, followed by incubation at −20°C for 20 min and centrifugation at 4°C for 10 min. The precipitate was dissolved by distilled water, if necessary, with incubation at 37°C.

The viral genome was digested with RQ1 DNase I (Promega) and Mung Bean Nuclease (Takara) according to the manufacturer’s instructions. The single-stranded DNA (ssDNA) (ФX174ss) and double-stranded DNA (dsDNA) (ФX174ds) from phiX174 phage (purchased from New England Biolabs) were used as controls. The nucleic acid concentrations were determined using a Nanodrop 1000 spectrophotometer (Thermo Fisher Scientific) and 1 μg was used for each reaction. RQ1 DNase I and Mung Bean Nuclease were used at 1 U and 0.025 U μg^−^^1^ DNA, respectively.

A previously reported degenerate primer mix (5′-ATGAATTCNNNNNNGATC-3′) (20) was used to amplify the viral genome DNA to acquire initial sequence data. Based on the initial data, six pairs of primers were designed (listed in Supplementary Table S2), and the polymerase chain reaction (PCR) products covering the whole genome were sequenced. The sequence data were assembled and analyzed using Vector NTI Advance 10 software. The open reading frames (ORFs) were searched and annotated, respectively, using the ORF_finder and PSI-BLAST programs at NCBI (http://www.ncbi.nlm.nih.gov). 

### Plasmid construction, gene knockout and complementation

Plasmids for gene knockout were constructed based on the suicide plasmid pHAR (21), and plasmids for gene complementation or spacer acquisition were constructed based on the expression plasmid pWL502, a pWL102 derivative with the mevinolin-resistant gene replaced by the *pyrF* gene (22) (Supplementary Table S1). Primers used for plasmid construction are listed in Supplementary Table S2.

The *cas* mutants and CRISPR variants were constructed using the previously described pop-in-pop-out strategy (21) with a few modifications. For the target *cas* gene(s), upstream and downstream fragments were separately amplified using corresponding UF/UR (upstream forward/upstream reverse) and DF/DR (downstream forward/downstream reverse) primer pairs, respectively, and then linked by bridge PCR with UF/DR primers. The linked fragments were inserted into the suicide plasmid pHAR to generate plasmids for *cas* knockout and validated by DNA sequencing. These plasmids were transformed into *H. hispanica* DF60 cells according to the Halohandbook online protocol (http://www.haloarchaea.com/resources/halohandbook/Halohandbook_2009_v7.2mds.pdf), and mutants were screened as previously described (21). Each *cas* mutant was validated by PCR analysis with UF/DR primers. The CRISPR variants were similarly constructed, except that a common UF/DR primer pair (DSP-UF and DSP-DR, Supplementary Table S2) was used for plasmid construction and mutant screening. 

Plasmids for *cas* complementation were constructed by cloning a fragment containing the *cas* operon promoter and the corresponding *cas* gene(s) into the expression vector pWL502. When necessary, the promoter and the encoding sequence were separately amplified with corresponding UF/UR and DF/DR primer pairs, and then linked by bridge PCR with UF/DR primers. A common UF primer (Comple_promoter-UF) was used. Alanine replacement of Cas3 residues was performed by PCR amplification with the plasmid template, a modified pGEM-T vector (pGEM-T Easy, Promega) bearing a wild-type Cas3-encoding gene. The PCR products were digested with DpnI and transformed into *E. coli* JM109 cells for cloning. Clones encoding Cas3 point mutants were screened by DNA sequencing.

The plasmid target pVS was constructed by cloning a 475-bp HHPV-2 fragment (genomic positions 7880–160) into pWL502. For the construction of other target plasmids (pCTC1-PLUS etc.), complementary oligonucleotides were annealed, forming sticky ends, and inserted into linearized pWL502 (digested by BamHI and KpnI).

### Spacer acquisition assay and protospacer analysis

To monitor spacer acquisition by PCR on virus infection, exponential *H. hispanica* culture was diluted 1:20 with fresh AS-168 medium, infected by HHPV-2 at a multiplicity of infection of 1 and cultured with shaking at 37°C until stationary stage. When necessary, serial sub-inoculations over several weeks were performed with repeated addition of fresh virus dilutions. For each PCR reaction, 100 ng genomic DNA was used as template. Primers are listed in Supplementary Table S2. Unless specified, oligonucleotides complementary to the leader (ExTest-LD) and spacer3 (ExTest-SP3) were used for DF60 and *cas* mutants, and oligonucleotides complementary to *cas2* (ExTest-CAS2) and spacer1 (ExTest-SP1) were used for CRISPR mutants.

Spacer acquisition from a plasmid target was similarly examined. The transformants were inoculated into yeast extract-subtracted AS-168 medium, cultured to exponential stage, subsequently transferred (1:100) into fresh AS-168 medium and then cultured until stationary stage. The stationary cultures were subjected to PCR analysis as described previously.

For protospacer analysis, the virus-infected or pVS-transformed DF60 culture was serially diluted and spread onto agar-containing AS-168 to obtain individual colonies. Colony PCR was performed to screen for clones containing an expanded CRISPR, which was subsequently analyzed by DNA sequencing. Based on the sequence information of new spacers, protospacers were identified by a Basic Local Alignment Search Tool (BLAST) search against the HHPV-2 genome or the plasmid sequence. The upstream sequence of each protospacer was collected and analyzed with Weblogo (http://weblogo.berkeley.edu/logo.cgi) to determine the PAM (protospacer adjacent motif).

## RESULTS

### Characterization of a new ssDNA halovirus infecting *H. hispanica*

**Figure 1.**
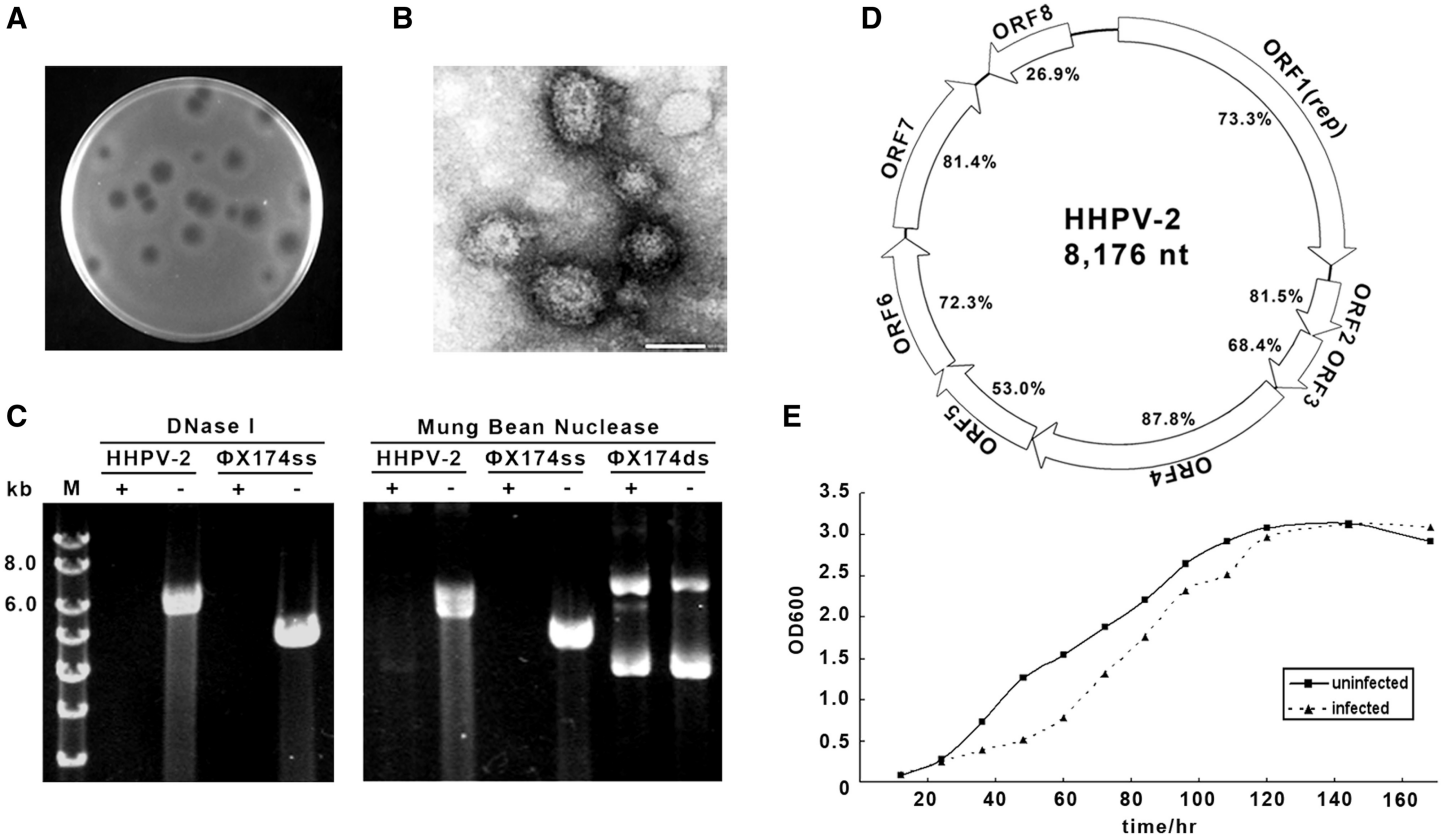
Isolation and characterization of a new halovirus HHPV-2. (**A**) HHPV-2 plaques on the *H. hispanica* lawn. (**B**) A representative transmission electron micrograph of negative-stained HHPV-2 virions. Bar, 50 nm. (**C**) DNase I (left) and Mung Bean Nuclease (right) digestions of the HHPV-2 genome, which proved to be an ssDNA molecule. The ssDNA genome (ФX174ss) and replicative form (ФX174ds) of phiX174 were also subjected to digestions. Lane M, dsDNA size marker. (**D**) A schematic depiction of the HHPV-2 genome. Similarities between HHPV-1 [a closely related dsDNA virus (23)] and HHPV-2 homologous gene products are shown. (**E**) Growth curve of uninfected (solid line) and virus-infected (dotted line) *H. hispanica* DF60 cultures.

The HHPV-2 virus was isolated from a hypersaline water sample from a saltern on Hulu Island (Liaoning, China). A single turbid plaque produced on the *H. hispanica* lawn (Figure 1A) was picked and inoculated into the liquid culture for enrichment. After virion purification and negative staining, transmission electron microscopy observations were performed (Figure 1B). The virions showed pleomorphic shapes, slightly elongated and without visible tail structures. The nucleic acid was extracted from the purified virions and subjected to nuclease digestions. Sensitivities to DNase I and the single strand-specific Mung Bean nuclease were observed (Figure 1C), indicating that the HHPV-2 genome is an ssDNA molecule. By genome sequencing, it was further revealed that the HHPV-2 genome is a circular molecule of 8176 nt and carries eight putative ORFs (Figure 1D). Interestingly, the genome organization shows a remarkable synteny to the genome of a dsDNA halovirus HHPV-1 (23), and polypeptides encoded by their ORFs share significant similarities (most between 70–80%). Therefore, it appears that HHPV-1 and HHPV-2 are closely related haloviruses, despite their different genome types (dsDNA and ssDNA, respectively) and sampling spots (Italy and China, respectively). Future investigations into the genetic determinants of their different replication behaviors may be interesting. Growth curve was plotted for HHPV-2-infected and -uninfected *H. hispanica* cultures (Figure 1E). A slightly retarded growth rate was observed for the infected culture, suggesting the viral progeny were released without cell lysis.

### New spacers were acquired discriminatively from the HHPV-2 genome

**Figure 2.**
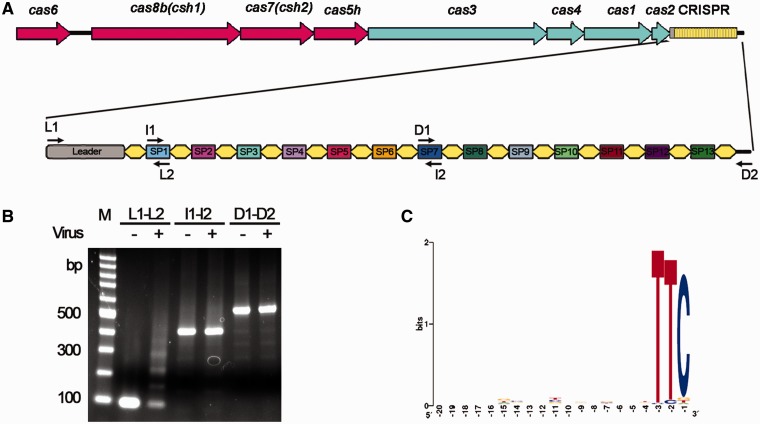
CRISPR adaptation to HHPV-2 infection. (**A**) Depiction of the single CRISPR structure and the preceding *cas* operon carried by the *H. hispanica* ATCC 33960 genome. Primers used to examine CRISPR expansion (in panel B) are shown as black arrows and listed in Supplementary Table S2. (**B**) PCR assay to detect CRISPR expansion at the leader end (L1–L2), the inner part (I1–I2) or the distal end (D1–D2). DNA sampled from infected (+) or uninfected (−) cells was used as PCR templates. Lane M, dsDNA size marker. (**C**) The sequence logo showing the conserved PAM of TTC. The 20 nt upstream of each protospacer observed during HHPV-2 infection were collected and analyzed with WebLogo (http://weblogo.berkeley.edu/logo.cgi).

The *H. hispanica* ATCC 33 960 genome carries a single CRISPR locus that is preceded by a *cas* operon consisting of four core *cas* genes (*cas1–4*) and four putative Cascade-encoding genes (*cas5–8*) (Figure 2A). To monitor spacer acquisition, three primer pairs were designed spanning the CRISPR array, and genomic DNA extracted from the virus-infected or -uninfected cells served as templates. Compared with the uninfected control, the infected sample produced larger PCR products only when the leader-proximal primers (L1–L2) were used (Figure 2B). Thus, it appeared that new spacers were inserted specifically at the leader-proximal end, whereas the inner part (I1–I2) and the distal end (D1–D2) were uninfluenced by virus infection. Using a titration experiment documented by Yosef *et al.* (12), our PCR procedure was proved able to detect CRISPR expansion in <1% cells (Supplementary Figure S1).

By plating the infected culture on agar medium, individual colonies showing CRISPR expansion were isolated and their PCR products were analyzed by DNA sequencing. For each individual colony, the higher molecular weight band proved to be a result of one or more spacer integration events (Supplementary Table S3). Notably, all the newly acquired spacers derived from the viral DNA, and none from the chromosome, indicating either a strict discrimination mechanism or the lethal effects of host-derived spacers. Apparently, this non-engineered CRISPR system adapted to HHPV-2 both efficiently and accurately. The newly acquired spacers confer host cells immunity to HHPV-2 reinfection (Supplementary Figure S2A). However, despite this immunity, subsequent spacer acquisition was still observed (Supplementary Figure S2B).

Nearly 94% (91 of 97) of the protospacers (from which new spacers were acquired) were preceded by a TTC PAM (Figure 2C), whereas the others were preceded by sequences showing high similarities to TTC (Supplementary Table S3). Therefore, the PAM sequence for this system seems to be a conserved TTC, which is consistent with a previous *in silico* analysis of *Haloquadratum walsbyi* spacers with metavirome data (18).

### Adaptation required Cas3 and Cascade protein(s)

**Figure 3.**
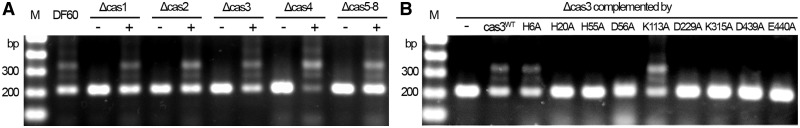
Adaptation to HHPV-2 infection under different *cas* genetic backgrounds. (**A**) Cas requirement for adaptation. For each *cas* mutant, DNA was sampled from cells transformed with an empty plasmid (−) or the plasmid carrying the deleted *cas* gene(s) (+). The plasmid-carried *cas* gene(s) was/were under the control of the *cas* operon promoter. (**B**) Requirements for the nuclease and helicase activities of Cas3. Alanine replacement was performed for the putative key residues in the HD nuclease domain (H20A, H55A, D56A and D229A) and the DExD/H helicase domain (K315A, D439A and E440A). Another two conserved residues (His6 and Lys113) were also mutated. The empty plasmid (−) and the plasmid carrying a wild-type Cas3 (Cas3^WT^) were used, respectively, as negative and positive controls. Lane Ms, dsDNA size markers.

In the *E. coli* type I-E system, Cas1 and Cas2 have been shown indispensable and sufficient for mediating naïve spacer acquisition (12). Single gene deletion mutants were constructed for *cas1–4* using the pop-in-pop-out strategy (Supplementary Figure S3A) (21). These mutants were infected by HHPV-2, and, interestingly, CRISPR expansion was not detected in any of the mutated strains even after serial sub-inoculations over several weeks (Figure 3A), suggesting adaptation also requires Cas3 and Cas4. To avoid potential polar effects, complementation was performed for each *cas* mutant with a plasmid carrying the corresponding single gene under the control of the *cas* operon promoter. As expected, CRISPR expansion was successfully rescued in these mutants (Figure 3A). For an unknown reason, we failed to construct single gene deletion mutants for the Cascade-encoding genes (*cas5–8*). However, a mutant (Δcas5–8) with the four genes simultaneously deleted was easily constructed (Supplementary Figure S3A and B). Similarly, Δcas5–8 cells did not acquire spacers until these genes were simultaneously complemented (Figure 3A), suggesting the important role of Cascade protein(s) during adaptation, though the requirement for each protein need be further investigated. Thus, in addition to Cas1 and Cas2, adaptation of this CRISPR system also required Cas3, Cas4 and at least partial Cascade proteins.

The RecB nuclease domain of Cas4 has been reported to be fused with Cas1 in some type I systems (9), which supports its involvement in adaptation. However, the requirements for Cascade protein(s) and Cas3 were unexpected because these proteins are involved in crRNA maturation and/or interference pathways (7). This reminded us that efficient adaptation observed here possibly was a primed process, whereas naïve adaptation may occur too infrequently to be detected by the above PCR analysis. Thus, we further screened 300 individual Δcas3 colonies, but none of them turned out able to acquire spacers (Supplementary Figure S4A). In contrast, >95% (100 of 105) of the *cas3*-complemented colonies acquired spacers efficiently (Supplementary Figure S4B). Therefore, the naïve adaptation pathway seemed inactivated in this system (also see later in the text).

It has been demonstrated *in vitro* that Cas3 possesses ssDNA nuclease and ATP-dependent helicase activities (24). Based on the multiple alignment of *H. hispanica* Cas3 and its most related homologs (Supplementary Figure S5), alanine replacement was performed for nine conserved residues, including putative key residues within the HD-type nuclease domain (His20, His55, Asp56 and Asp229) and the DExD/H-box helicase domain (Lys315, Asp439 and Glu440). As expected, cells producing Cas3 point mutants with these key residues replaced failed to acquire spacers (Figure 3B). Because the activity of one domain has been shown insusceptible to mutations in the other (24), the nuclease and helicase activities seemed both indispensible for adaptation, reaffirming the requirement for the interference machinery and suggesting a priming process mediated by some preexisting spacer(s).

### Adaptation need be primed by a preexisting spacer partially matching the invader DNA

**Figure 4.**
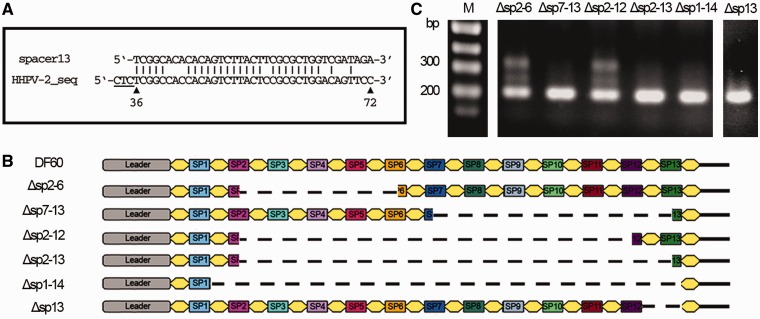
A preexisting spacer (spacer13) matching the HHPV-2 genome was required. (**A**) Depiction of the imperfect match between spacer13 and an HHPV-2 fragment. Positions of the fragment on the HHPV-2 genome are indicated under the sequence. Three upstream nucleotides corresponding to PAM motif are underlined. (**B**) Depiction of the spacer content of CRISPR variants. Δsp2–6, Δsp7–13, Δsp2–12, Δsp2–13 and Δsp1–14 are designated according to the spacers (or repeat) bordering the omitted region (dashed lines). In Δsp13, spacer13 and the immediately upstream repeat were deleted. (**C**) Adaptation of variant CRISPRs to HHPV-2 infection. Lane M, dsDNA size marker.

We performed a BLAST search against the HHPV-2 genome using the preexisting spacers as query sequences, and spacer13, the most distal one from the leader, showed the highest identity (∼73%) to an HHPV-2 fragment (Figure 4A). To determine whether this partial complementarity have facilitated adaptation, a series of CRISPR mutants were constructed with their spacer content partially omitted (Figure 4B; Supplementary Figure S3C). When infected, cells (Δsp2–6 and Δsp2–12) retaining spacer13 showed evident CRISPR expansion, whereas those with spacer13 deleted (Δsp1–14 and Δsp13) or truncated (Δsp7–13 and Δsp2–13) failed to acquire spacers (Figure 4C), suggesting the requirement for a priming process mediated by this spacer.

For mutants whose priming adaptation was blocked by deleting/truncating the priming spacer, naïve adaptation was supposed to be unaffected. Given the knowledge that naïve adaptation stimulates subsequent priming adaptation during a prolonged cultivation (13,14), we serially sub-inoculated the CRISPR mutants over six weeks, with repeated addition of fresh virus dilutions, to detect the rescue of the priming pathway by potential naïve events. However, a similar result to Figure 4C was observed, indicating the inactivation of naïve adaptation in this system, which is also supported by the Δcas3 colony screening result (Supplementary Figure S4).

**Figure 5.**
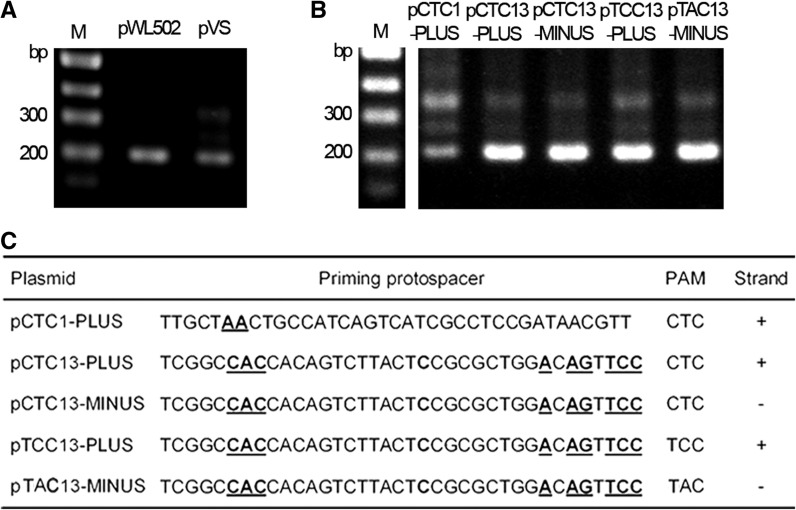
Adaptation to engineered plasmids. (**A**) Adaptation to a modified pWL502 plasmid (pVS) carrying a viral sequence that is partially matched by spacer13. The empty plasmid (pWL502) was used as a control. (**B**) Adaptation to modified pWL502 plasmids carrying different artificial priming protospacers. (**C**) Information of the engineered plasmids in panel B. The plasmid designations indicate their PAM sequences (CTC, TCC or TAC) and different priming protospacers (partially matched by spacer1 or spacer13) on different strands (+ or −). Within the priming protospacer sequence, nucleotides that were designed mismatching the corresponding spacer are shown in bold and underlined. The plus (+) and minus (−) strands correspond, respectively, to the coding and template strands of the *pyrF* gene. These engineered plasmids were transformed into wild-type DF60 cells and examined for CRISPR expansion. Land Ms, dsDNA size markers.

A plasmid target (pVS) bearing a viral fragment containing the priming protospacer of spacer13 was constructed. Remarkably, efficient adaptation was observed when the wild-type DF60 cells were transformed by pVS, but not by the control plasmid (Figure 5A). We further constructed plasmids carrying different priming protospacers (partially matched by spacer1 or spacer13) on different strands (pCTC1-PLUS, pCTC13-PLUS and pCTC13-MINUS) (Figure 5C), to which CRISPR adaptation was consistently observed (Figure 5B). We also changed the PAM sequence to TCC (pTCC13-PLUS) or TAC (pTAC13-MINUS), which appeared not to block adaptation (Figure 5B). However, more PAM sequences need be tested to access the role of PAM during priming adaptation. Apparently, these combined results underlined the essential role of a priming process during adaptation to different invaders.

### Spacer acquisition showed a strand bias directed by the priming spacer

**Figure 6.**
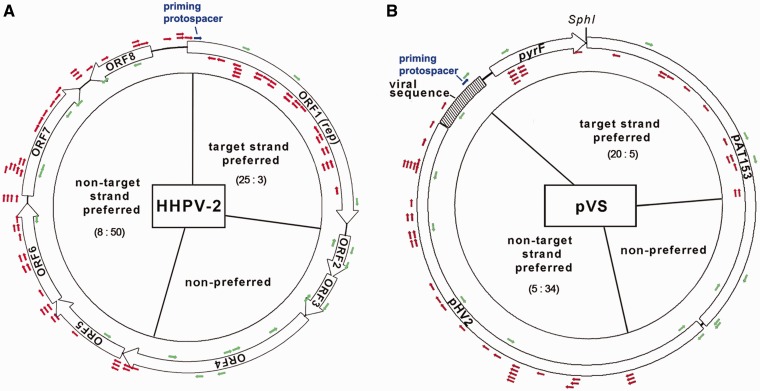
Spacer acquisition shows a strand bias directed by the priming spacer. The priming protospacer, which is imperfectly targeted by the priming spacer (spacer13), is shown as a blue arrow. Protospacers from which new spacers were acquired during HHPV-2 infection (**A**) and pVS transformation (**B**) are shown as red or green arrows. Red arrows indicate protospacers located on the preferred strand, whereas the green ones indicate those on the non-preferred strand or within the non-preferred genomic region. For each region, the ratio of protospacers on the two strands (target versus non-target) is given. For HHPV-2, the double-stranded replication form genome is shown. The plasmid pVS carries an HHPV-2 fragment (shadowed) containing the priming protospacer. The crRNA of spacer13 is expected to base pair to the target strand of the priming protospacer (the template strand of the *rep* or *pyrF* gene) and displace the non-target strand (the coding strand of the *rep* or *pyrF* gene).

Priming adaptation exhibits a strand bias in *E. coli* (13,14); thus, a similar strand bias is expected if adaptation to HHPV-2 is primed by spacer13. We obtained 97 virus-derived spacers from 56 randomly selected colonies showing CRISPR expansion (Supplementary Table S3) and mapped them onto the viral genome. Unexpectedly, different strand bias between DNA sequences upstream and downstream of the priming spacer was observed (Figure 6A). According to a previous study (25), the crRNA of spacer13 is expected to base pair to the target strand (the template strand of the *rep* gene) and displace the non-target strand (the coding strand of the *rep* gene) of the priming protospacer. Nearly 86% (50/58) of the upstream protospacers are located on the non-target strand of the priming protospacer, whereas ∼89% (25/28) of the downstream ones on the target strand. The preferred orientations of upstream and downstream protospacers, respectively, match and counter to the orientation of the priming protospacer. Only 11% (11/97) of the protospacers resided within genome positions 2279–4400 (which are equal to 1/4 of the genome and distant from the priming protospacer), and thus strand preference could not be identified. Besides, spacer acquisition from the non-target strand upstream of the priming protospacer seemed more efficient (50:25) than from the downstream target strand.

A similar preference pattern was observed during adaptation to the plasmid target pVS (Figure 6B; Supplementary Table S4). The distribution of the theoretical PAM motif (TTC) on HHPV-2 genome and pVS showed no evident strand bias (Supplementary Table S5), thus the spacer acquisition strand bias could not have derived from uneven PAM distribution. Given this knowledge and the different DNA sequences of HHPV-2 and pVS, an intrinsic mechanism is expected to determine the observed preference pattern.

### Bioinformatic analysis of the potential priming spacers in the *S. thermophilus* system

**Figure 7.**
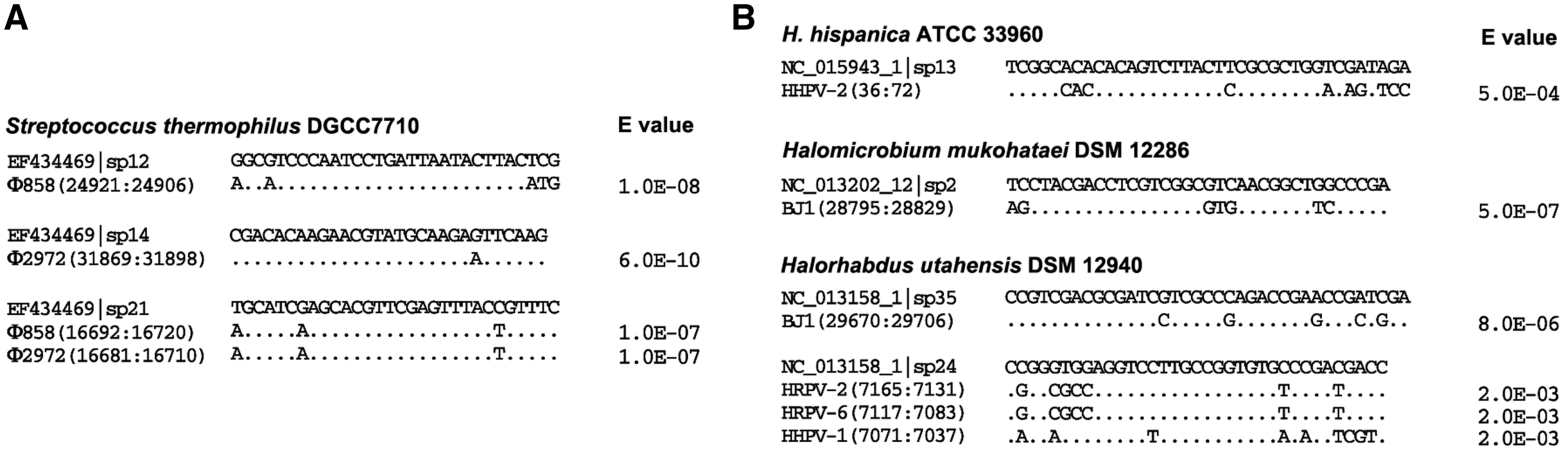
Imperfect matches between preexisting spacers and viral (or phage) DNA. (**A**) *S. thermophilus* DGCC7710 CRISPR1 (under the GenBank accession number EF434469) contains preexisting spacers matching the Ф858 and Ф2972 genomes. (**B**) Representative matches between haloarchaeal spacers and haloviral genomes. The CRISPR IDs used in the CRISPRdb database (http://crispr.u-psud.fr/crispr/) are given. The match between *H. hispanica* spacer13 and HHPV-2 is also shown as a reference. The *E*-value of each match is shown. Dots in the viral (or phage) sequences indicate nucleotides identical to spacers at these positions. The genome positions of the viral (or phage) sequences are indicated by two numbers separated by a colon.

Efficient adaptation mediated by a natural type II-A CRISPR system has been previously reported in *S. thermophilus* (10), which is the first and, so far as we know, the only previously reported efficient adaptation of a native CRISPR to purified viruses. We noted that the *S. thermophilus* DGCC7710 wild-type CRISPR1 locus (under the GenBank accession number EF434469) contains 32 preexisting spacers before infection. With these spacers as query sequences, a BLAST search was performed against the genomes of Ф858 and Ф2972 phages, to which CRISPR adaptation was observed. Interestingly, the preexisting spacer12 partially matches to the Ф858 genome, spacer14 to the Ф2972 genome and spacer21 to a conserved sequence from both genomes (Figure 7A). Each match exhibits a higher similarity than that between *H. hispanica* spacer13 and HHPV-2 genome. Thus, the possibility is considerable that these preexisting spacers may have contributed to the previously reported adaptation via the priming pathway.

In addition, a BLAST search with 720 spacer sequences (from nine haloarchaeal strains) was performed against 13 available haloviral genomes. Despite the limited virus information, five strains (*Halorubrum lacusprofundi* ATCC 49239, *Halomicrobium mukohataei* DSM 12286, *Halorhabdus utahensis* DSM 12940, *Natrialba magadii* ATCC 43099 and *H. hispanica* ATCC 33 960) contain spacers partially matching one or more viral genomes with an E value < 0.001 (Supplementary Table S6). The other strains also carry spacers with lower similarities (E value < 0.01) to haloviral sequences. Representative matches are shown in Figure 7B. It was implied that some of these spacers may be able to prime CRISPR adaptation to the targeted haloviruses, which needs to be tested by future studies on more host–virus interactions.

## DISCUSSION

Bioinformatic and metagenomic studies have demonstrated the highly dynamic feature of the CRISPR-Cas system (26–28), which confers on host cells an adaptive immunity against numerous invaders. This adaptability is believed to be derived from the frequent acquisition of short DNA fragments from new invaders. Curiously, since the first report of efficient adaptation to purified viruses in *S. thermophilus* in 2007 (10), such adaptation has been rarely reported for other host–invader interactions, except when *cas* genes were overexpressed (12), CRISPR was engineered (13) or an environmental virus mixture was used for infection (15). Studies on the engineered *E. coli* type I-E CRISPR have introduced the concept of two different adaptation pathways, i.e. naïve adaptation and priming adaptation (13,14).

In this study, we characterized another host–virus interaction that shows efficient spacer acquisition. Intriguingly, this process definitely required not only Cas1 and Cas2 but also other Cas proteins (those involved in crRNA maturation or/and interference), as well as a preexisting spacer (spacer13) partially matching the viral DNA. These genetic requirements raised the hypothesis that the observed adaptation was primed by this spacer. Efficient adaptation was reproduced to an engineered plasmid bearing the spacer13-partially-matched viral fragment. During the virus- and the plasmid-based invader assays, spacer acquisition consistently exhibited a strand preference pattern directed by the priming spacer. These combined results reinforced the judgment of the observed adaptation to be a primed process. Regarding that the virus HHPV-2 and the host strain *H. hispanica* ATCC 33 960 were isolated from salterns in China and Spain (29), respectively, it is surprising to see the adaptation primed by such an ancient spacer (at the distal end).

We further investigated the case in *S. thermophilus*, and found preexisting spacers partially complementary to the Ф858 and Ф2972 genomes (Figure 7A), with much smaller *E*-values than the complementarity between the *H. hispanica* priming spacer (spacer13) and HHPV-2 genome. Though the requirements for these spacers need to be further tested by genetic studies, the possibility of their priming roles in the previously reported adaptation (10) was considerable. Thus, we speculate that the two so far characterized efficient adaptation models (a native type II-A or I-B system, respectively) both have the potential to trigger a priming process with their preexisting spacers. This persuaded us to speculate that the failures to observe efficient adaptation in many laboratory strains may be attributed to the lack of preexisting priming spacers. One may argue that priming spacers could be provided by naïve adaptation, which has been shown inefficiently initiated in the engineered type I-E system (13,14). However, in the *H. hispanica* system, the naïve pathway seems inactivated because CRISPR expansion was not detected when the priming pathway was blocked by removing the priming spacer even after serial sub-inoculations with repeated virus infections (during which a new spacer acquired via the naïve pathway is expected to restore the priming pathway).

The inactivation of naïve adaptation in our system is surprising because naïve adaptation is a prerequisite for any subsequent primed processes, and CRISPR adaptability will be compromised especially when encountering a new virus. However, in the view of self-immunity, inactivation of the naïve pathway is reasonable and necessary, especially for laboratory strains. During long-term virus-free culturing, the host DNA of laboratory strains has served as the only DNA sources, in this case, naïve adaptation could be detrimental because it does not discriminate between non-self versus self DNA well (12–14), and host-derived spacers will lead to toxic effects or even cell death in the presence of an active interference pathway. Thus, we speculate that, for some, if not all, laboratory strains, naïve adaptation may have been inactivated or the whole system been silenced by, for example, H-NS (30,31), which may underlie the vacancy of adaptation reports for these systems.

In a recent study on *S. solfataricus* CRISPR system, adaptation was observed to an environmental virus mixture, but curiously, not to a single purified virus (STSV2) (15). This difference may be attributed to the requirement of a priming process. The environmental sample contains much higher DNA sequence diversity than the purified virus, thus has a greater potential to trigger the priming pathway. One could speculate that the newly acquired spacers targeting a conserved DNA sequence are able to prime adaptation to other related invaders. From this perspective, even without the naïve pathway, priming adaptation seems powerful enough to provide an adaptive immunity to invaders in natural environments. However, we should admit that naïve adaptation is essential to shape the initial CRISPR memory.

**Figure 8.**
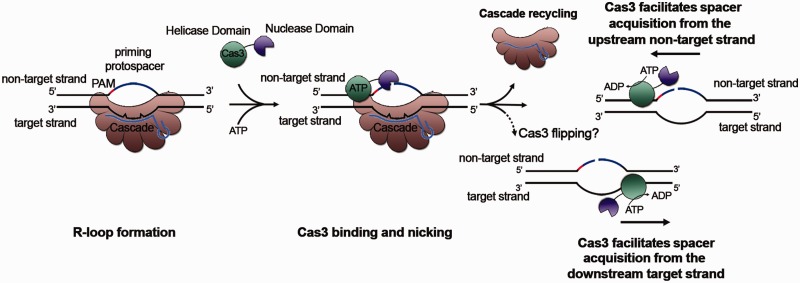
A possible priming model explaining the strand bias changes between the DNA sequences upstream and downstream of the priming protospacer. Despite several mismatches between the crRNA of the priming spacer and the target DNA, Cascade still binds to the latter with a reduced affinity, forming the R-loop. Cas3 is then recruited to the non-target strand and nicks it. In a possible Cascade replacement process, *H. hispanica* Cas3 may occasionally flip onto the target strand via an unknown mechanism. The Cas3 helicase (or translocase) activity may facilitate the 3′–5′ movement of the adaptation machinery on the upstream non-target strand or on the downstream target strand of the priming protospacer.

Notably, the spacer acquisition preference pattern observed in this study suggests that the adaptation machinery, assembled at the priming protospacer, moves along the target or the non-target strand, consistently in the 3′–5′ direction, which is reminiscent of the reported 3′–5′ directionality of Cas3 helicase (or perhaps DNA translocase) activity (24). In *E. coli*, the movement of the adaptation machinery appears also in the 3′–5′ direction, but specifically on the non-target strand (13,14). Thus, there must be a different mechanism allowing *H. hispanica* Cas3 binding to the target strand of the priming protospacer. Based on recent studies on Cas3 (24,32), we hypothesize that *H. hispanica* Cas3 may occasionally flip from the non-target strand to the target strand during Cascade replacement via unknown mechanisms (Figure 8).

In summary, adaptation of this haloarchaeal type I-B system was proved to be a priming process, and the naïve pathway seemed inactivated. The universality of this priming requirement needs to be tested by future studies on more CRISPR systems of other subtypes. These studies will be of great significance because they will help to explain the discrimination mechanism at the adaptation level, which has confused scientists for years.

## ACCESSION NUMBERS

The HHPV-2 viral genome sequence was deposited in the GenBank database under the accession number KF056323.

## SUPPLEMENTARY DATA

Supplementary Data are available at NAR Online.

## FUNDING

National Natural Science Foundation of China [30925001, 31271334, and 31330001]. Funding for open access charge: National Natural Science Foundation of China [30925001].

*Conflict of interest statement*. None declared.

## Supplementary Material

Supplementary Data
