# Supplementary material for: Adaptation of the Haloarcula hispanica CRISPR-Cas system to a purified virus strictly requires a priming process
Source: Nucleic Acids Res. 2013 Nov 20;42(4):2483–92. doi: 10.1093/nar/gkt1154 (PMC3936756; doi:10.1093/nar/gkt1154)
Supplement: Supplementary Data [file supp_42_4_2483__index.html]

Adaptation of the Haloarcula hispanica CRISPR-Cas system to a purified virus strictly requires a priming process — Adaptation of the Haloarcula hispanica CRISPR-Cas system to a purified virus strictly requires a priming process — Supplementary Data 

# Adaptation of the *Haloarcula hispanica* CRISPR-Cas system to a purified virus strictly requires a priming process

## Supplementary Data

files

**Files in this Data Supplement:**

- Supplementary Data - pdf file
