# Supplementary material for: Adaptation of the Haloarcula hispanica CRISPR-Cas system to a purified virus strictly requires a priming process
Source: Nucleic Acids Res. 2013 Nov 20;42(4):2483–92. doi: 10.1093/nar/gkt1154 (PMC3936756; doi:10.1093/nar/gkt1154)
Supplement: Supplementary Data [file supp_gkt1154_nar-02405-h-2013-File010.pdf]

SUPPLEMENTARY DATA FOR

Adaptation of the *Haloarcula hispanica* CRISPR-Cas system to a purified virus strictly requires a priming process

Ming Li[1,2], Rui Wang[1,2], Dahe Zhao[1] and Hua Xiang[1]

[1]*State Key Laboratory of Microbial Resources,*

Institute of Microbiology, Chinese Academy of Sciences, Beijing, China.

[2]*University of Chinese Academy of Sciences, Beijing, China*

Summary of supplementary figures and tables:

Figure S1. Detection limit of PCR assay to monitor CRISPR expansion

Figure S2. Physiological effects of the new spacers on host cells

Figure S3. Validation of the genotype of *cas* mutants and CRISPR variants

Figure S4. Validation of the requirement of Cas3 for adaptation by colony screening

Figure S5. Multiple alignment of *H. hispanica* Cas3 and its most related homologues

Table S1. Strains and plasmids used in this study

Table S2. Oligonucleotides used in this study

Table S3. Spacers acquired during HHPV-2 infection assay

Table S4. Spacers acquired during pVS (a plasmid carrying a viral sequence) transformation assay

Table S5. Distribution of the theoretical PAM (TTC) and the observed protospacers on the HHPV-2 genome or the target plasmid pVS

Table S6. Imperfect matches between haloarchaeal spacers and haloviral genomes

SUPPLEMENTARY FIGURES

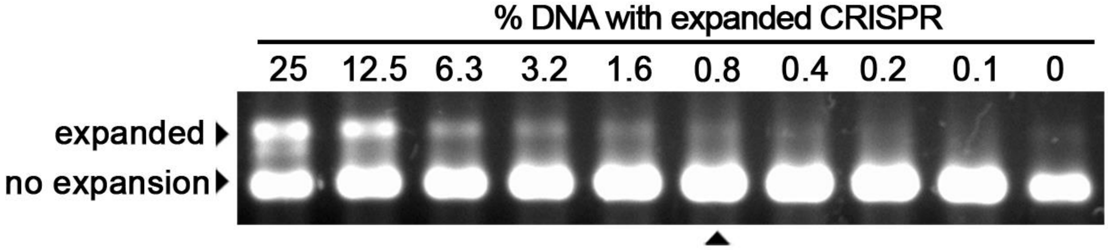

Figure S1. Detection limit of PCR assay to monitor CRISPR expansion. Two DNA samples, extracted respectively from *H. hispanica* cells with (expanded) and without (no expansion) a newly acquired spacer, were diluted to the same concentration and mixed at a serial of ratios (the percentages of the expanded sample are shown above). The detection limit is indicated with an arrowhead at the bottom. Three independent experiments showed similar results to this gel.

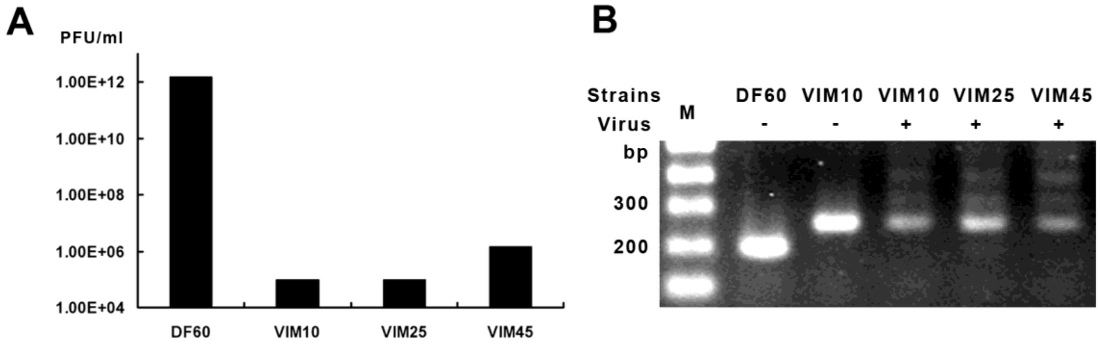

Figure S2. Physiological effects of the new spacers on host cells. (A) Virus resistance was provided by new spacers, with a 10^6- to 10^7-fold drop in PFU/ml during plaque assay. We selected three individual colonies (VIM10, VIM25, and VIM45) each containing one new spacer for analysis. VIM, virus insensitive mutant. **(B)** More new spacers were acquired during HHPV-2 re-infection. M, dsDNA size marker.

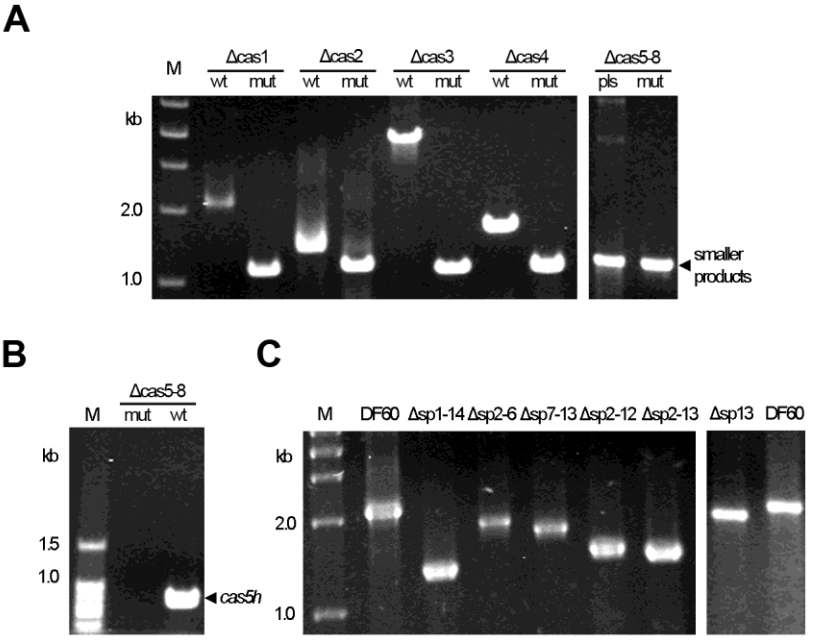

Figure S3. Validation of the genotype of *cas* mutants and CRISPR variants. (A) PCR analysis was performed against genomic DNA from each *cas* mutant (mut) with the UF/DR primers. For Δcas1, Δcas2, Δcas3, and Δcas4 mutants, genomic DNA from DF60 was used as a negative control (wt), while for Δcas5-8, the knockout plasmid pDCAS5-8 was also subjected to PCR amplification as a positive control (pls). The smaller products (~1.2 kb) indicate the absence of the target *cas* gene(s). **(B)** Validation of the Δcas5-8 mutant (mut) by confirming the absence of the *cas5h* gene. DF60 genomic DNA was subjected to PCR amplification as a positive control (wt). The primer pair Cas5h-test-F/R was used. **(C)** PCR analyses against genomic DNA from DF60 and CRISPR variants with the DSP-UF/DSP-DR primers. The wild-type CRISPR in DF60 generated a 2.10-kb PCR product, and PCR products with smaller MWs were produced from the variant CRISPRs of Δsp1-14 (~1.31 kb), Δsp2-6 (~1.83 kb), Δsp7-13 (~1.71 kb), Δsp2-12 (~1.45 kb), Δsp2-13 (1.38 kb), and Δsp13 (~2.03 kb). Lane Ms, dsDNA size markers. The PCR primers are listed in Supplementary Table S2.

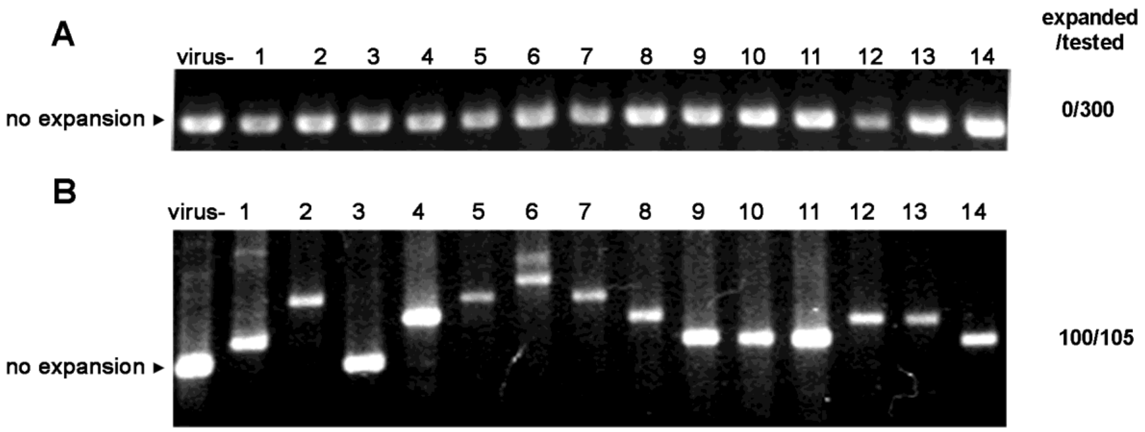

Figure S4. Validation of the requirement of Cas3 for adaptation by colony screening. Representative gels showing the screening of 300 Δcas3 colonies (**A**) and 105 *cas3*-complemented colonies (**B**) for expanded CRISPRs. The DNA sample from uninfected culture (virus-) was amplified as a control. The ratios of colonies showing CRISPR expansion are indicated on the right.

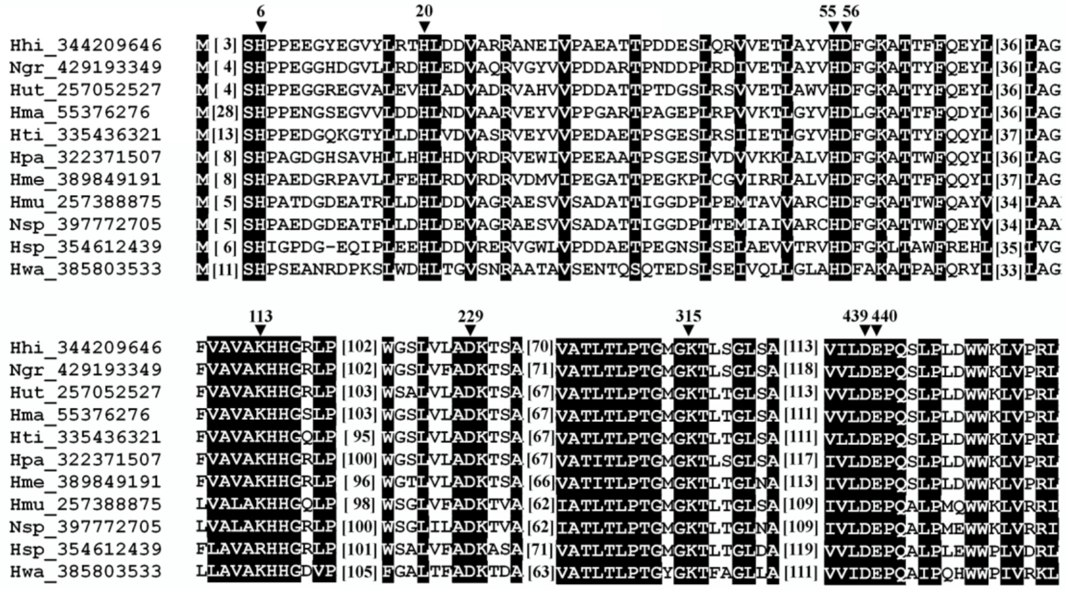

Figure S5. Multiple alignment of *H. hispanica* Cas3 and its most related homologues. The conserved residues are shown in white against black background. The residues replaced by alanine for point mutation were indicated with black arrows and their positions within *H. hispanica* Cas3 sequence. His20, His55, and Asp56 are putative key residues in the HD-type nuclease domain, while Asp229, Lys315, Asp439, and Glu440 are putative key residues in the DExD/H helicase domain (1). Numbers in brackets indicate the length of unshown interval sequences. The source-organism abbreviation and GenBank identifier are shown on the left column. Hhi, *Haloarcula hispanica*; Ngr, *Natronobacterium gregoryi*; Hut, *Halorhabdus utahensis*; Hma, *Haloarcula marismortui*; Hti, *Halorhabdus tiamatea*; Hpa, *Haladaptatus paucihalophilus*; Hme *Haloferax mediterranei*; Hmu, *Halomicrobium mukohatae*; Nsp, *Natrinema* sp. J7-2; Hsp, *Halobacterium* sp. DL1; Hwa, *Haloquadratum walsbyi*.

SUPPLEMENTARY TABLES

Table S1. Strains and plasmids used in this study

Strains/Plasmids	Description	Source or reference
***H. hispanica* strains**		
DF60	*pyrF*-deleted strain of *H. hispanica* ATCC 33960	(2)
Δcas1	*cas1*-deleted mutant of DF60	This study
Δcas2	*cas2*-deleted mutant of DF60	This study
Δcas3	*cas3*-deleted mutant of DF60	This study
Δcas4	*cas4*-deleted mutant of DF60	This study
Δcas5-8	Cascade-encoding genes (*cas5-8*) deleted mutant of DF60	This study
Δcas1::1	Δcas1 complemented with a plasmid-carried *cas1*	This study
Δcas2::2	Δcas2 complemented with a plasmid-carried *cas2*	This study
Δcas3::3	Δcas3 complemented with a plasmid-carried *cas3*	This study
Δcas4::4	Δcas4 complemented with a plasmid-carried *cas4*	This study
Δcas5-8::5-8	Δcas5-8 complemented with plasmid-carried *cas5-8* genes	This study
Δcas3::H6A	Δcas3 complemented with a $Cas3^{H6A}$-encoding gene	This study
Δcas3::H20A	Δcas3 complemented with a $Cas3^{H20A}$-encoding gene	This study
Δcas3::H55A	Δcas3 complemented with a $Cas3^{H55A}$-encoding gene	This study
Δcas3::D56A	Δcas3 complemented with a $Cas3^{D56A}$-encoding gene	This study
Δcas3::K113A	Δcas3 complemented with a $Cas3^{K113A}$-encoding gene	This study
Δcas3::D229A	Δcas3 complemented with a $Cas3^{D229A}$-encoding gene	This study
Δcas3::K315A	Δcas3 complemented with a $Cas3^{K315A}$-encoding gene	This study
Δcas3::D439A	Δcas3 complemented with a $Cas3^{D439A}$-encoding gene	This study
Δcas3::E440A	Δcas3 complemented with a $Cas3^{E440A}$-encoding gene	This study
Δsp2-6	DF60 with spacer2-6 deleted or truncated	This study
Δsp7-13	DF60 with spacer7-13 deleted or truncated	This study
Δsp2-12	DF60 with spacer2-12 deleted or truncated	This study
Δsp2-13	DF60 with spacer2-13 deleted or truncated	This study
Δsp1-14	DF60 with spacer2-13 completely deleted	This study
**Plasmids**		
pHAR	4.0 kb; suicide vector containing *pyrF* and its native promoter	(2)
pWL502	7.9 kb; expression vector containing a *pyrF* and its native promoter derived from *Haloferax mediterranei*	(3)
pDCAS1	5.1 kb; modified pHAR for knockout of *cas1*	This study
pDCAS2	5.1 kb; modified pHAR for knockout of *cas2*	This study
pDCAS3	5.2 kb; modified pHAR for knockout of *cas3*	This study
pDCAS4	5.1 kb; modified pHAR for knockout of *cas4*	This study
pDCAS5-8	5.1 kb; modified pHAR for knockout of *cas5-8*	This study
pDSP2-6	5.8 kb; modified pHAR for knockout of spacer2-6	This study
pDSP7-13	5.7 kb; modified pHAR for knockout of spacer7-13	This study
pDSP2-12	5.5 kb; modified pHAR for knockout of spacer2-12	This study

pDSP2-13	5.4 kb; modified pHAR for knockout of spacer2-13	This study
pDSP1-14	5.3 kb; modified pHAR for removal of spacer2-13	This study
pDSP13	6.0 kb; modified pHAR for clean removal of the 13th repeat-spacer unit	This study
pCAS1	9.4 kb; modified pWL502 carrying *cas1* and the *cas* operon promoter	This study
pCAS2	8.9 kb; modified pWL502 carrying *cas2* and the *cas* operon promoter	This study
pCAS3	11.0 kb; modified pWL502 carrying *cas3* and the *cas* operon promoter	This study
pCAS4	8.9 kb; modified pWL502 carrying *cas4* and the *cas* operon promoter	This study
pCAS5-8	13.5 kb; modified pWL502 carrying *cas5-8* and the *cas* operon promoter	This study
pH6A	11.0 kb; mutated pCas3 encoding Cas3^{H6A}	This study
pH20A	11.0 kb; mutated pCas3 encoding Cas3^{H20A}	This study
pH55A	11.0 kb; mutated pCas3 encoding Cas3^{H55A}	This study
pD56A	11.0 kb; mutated pCas3 encoding Cas3^{D56A}	This study
pK113A	11.0 kb; mutated pCas3 encoding Cas3^{K113A}	This study
pD229A	11.0 kb; mutated pCas3 encoding Cas3^{D229A}	This study
pK315A	11.0 kb; mutated pCas3 encoding Cas3^{K315A}	This study
pD439A	11.0 kb; mutated pCas3 encoding Cas3^{D439A}	This study
pE440A	11.0 kb; mutated pCas3 encoding Cas3^{E440A}	This study
pVS	8.3 kb; modified pWL502 carrying a viral sequence	This study
pCTC1-PLUS	7.9 kb; modified pWL502 carrying a priming protospacer	This study
pCTC13-PLUS	7.9 kb; modified pWL502 carrying a priming protospacer	This study
pCTC13-MINUS	7.9 kb; modified pWL502 carrying a priming protospacer	This study
pTCC13-PLUS	7.9 kb; modified pWL502 carrying a priming protospacer	This study
pACC13-MINUS	7.9 kb; modified pWL502 carrying a priming protospacer	This study

Table S2. Oligonucleotides used in this study

**Primer**[a]	**5'–3' sequence**[b]
**For viral genome sequencing**	
genome-seq-1F	CGGGACTATCTGTCACACTG
genome-seq-1R	GCCGCGCTCGCTCTTTATCCTGA
genome-seq-2F	CATGCCATGGCGGCGCGACTGTTGTAATGAAGA
genome-seq-2R	GGGGCAACAGCAGAATCAGGTAT
genome-seq-3F	CAGTCCGTTGCTTCGTATGCTGC
genome-seq-3R	CGAGGAAGGCGATCTGCATAC
genome-seq-4F	CGCTGCGTTCGCCTCGTCTGA
genome-seq-4R	GATACCTCTATTTACCGAAC
genome-seq-5F	GCAGAACATTCTGACTCATT
genome-seq-5R	TCAGCTCATACGACCGAGAC
genome-seq-6F	TCGGGTCCGGTCTGATC
genome-seq-6R	CGAACAGACGACGATTCAGT
**For spacer acquisition assay**	

ExTest-L1	CCGCCGTTAGCAGCCTTAT
ExTest-L2	TCGGAGGCGATGACTGAT
ExTest-I1	CAGTCATCGCCTCCGATAA
ExTest-I2	GCGGCGGTCTTGTTCA
ExTest-T1	CGGCGGGAGCATGAAC
ExTest-T2	GCGGCGACGGTTGAGTATAT
ExTest-LD	CCCGCCGTTAGCAGCCTTAT
ExTest-SP3	CCGCCGGCAATGACGTT
ExTest-CAS2	TACGGCGACGATCCAACTGA
ExTest-SP1	CGGAGGCGATGACTGATG
**For *cas* gene knockout**	
DCAS1-UF	CGCGGATCCCTGAGAGGCGGATCTTATGA
DCAS1-UR	**CATCACCACGTACATTGTTAACACCAACAG**AAGTCAT
DCAS1-DF	**CTGTTGGTGTTAACAATGTACGTGGTGATG**GTATAC
DCAS1-DR	ATAGGGTACCGCCGGCAATGACGTTC
DCAS2-UF	CGCGGATCCGTCTCCCGATTGACGAACT
DCAS2-UR	**AACCGCTGATCCTCATTTACCACCACCGTT**GGAA
DCAS2-DF	**AACGGTGGTGGTAAATGAGGATCAGCGGTT**TCTATA
DCAS2-DR	ATAGGGTACCGGCGGTCTTGTTCATGCT
DCAS3-UF	CGCGGATCCGGGAGATTAGGACGGTCAA
DCAS3-UR	**GATCTGCATCGGTCATCAGACGAACACCAC**GGTT
DCAS3-DF	**GTGGTGTTCGTCTGATGACCGATGCAGATC**CAGT
DCAS3-DR	ATAGGGTACCCATGGTACGCACAGGAGTCA
DCAS4-UF	CGCGGATCCTGCAGAGGCGGTCTACAAT
DCAS4-UR	**AGTTCCTATCCATTGTCATAAGATCCGCCT**CTCA
DCAS4-DF	**AGGCGGATCTTATGACAATGGATAGGAACT**ACCACGT
DCAS4-DR	ATAGGGTACCCGTTGGGAGAGCTGGAACT
DCAS6875-UF	CGCGGATCCCGAGCAAGGCGCAATAGAT
DCAS6875-UR	**AGAGTACATAGTCATTACACGCACTCGGATG**ACTCA
DCAS6875-DF	**CATCCGAGTGCGTGTAATGACTATGTACTCT**CATCCA
DCAS6875-DR	ATAGGGTACCCACGATCCATCGTTATCAGTT
Cas5h-test-F	ATGTCACAACAATCGCT
Cas5h-test-R	TCATCAGACGAACACCA
**For *cas* gene complementation**	
Comple_promoter-UF	CGCGGATCCGGGGCACGGTCGATTCTC
Comple_CAS1-UR	**GGTAGTTCCTATCCATTACACGCACTCGGAT**GACTC
Comple_CAS1-DF	**ATCCGAGTGCGTGTAATGGATAGGAACTACC**ACGT
Comple_CAS1-DR	ATAGGGTACCTTACCACCACCGTTGGAA
Comple_CAS2-UR	**CATCACCACGTACATTACACGCACTCGGAT**GACTC
Comple_CAS2-DF	**ATCCGAGTGCGTGTAATGTACGTGGTGATG**GTATA
Comple_CAS2-DR	ATAGGGTACC CTATAGAAACCGCTGAT
Comple_CAS3-UR	**AGAGTACATAGTCATTACACGCACTCGGAT**GACTC
Comple_CAS3-DF	**ATCCGAGTGCGTGTAATGACTATGTACTCT**CATCC
Comple_CAS3-DR	ATAGGGTACCTCATAAGATCCGCCTCTCAG

Comple_CAS4-UR	**ATCTGCATCGGTCATTACACGCACTCGGAT**GACTC
Comple_CAS4-DF	**ATCCGAGTGCGTGTAATGACCGATGCAGAT**CCAGT
Comple_CAS4-DR	ATAGGGTACCTTAACACCAACAGAAGTC
Comple_CAS6875-F	CGCCCATGGGGGGCACGGTCGATTCTC
Comple_CAS6875-R	ATAGGGTACCTCAGACGAACACCACGGTT
**For Cas3 point mutagenesis**	
CAS3-H6A-F	ATGACTATGTACTCTGCTCCACCCGAAGAGGGC
CAS3-H6A-R	GCCCTCTTCGGGTGGAGCAGAGTACATAGTCAT
CAS3-H20A-F	GTCTATCTTCGGACGGCTCTGGATGATGTTGCC
CAS3-H20A-R	GGCAACATCATCCAGAGCCGTCCGAAGATAGAC
CAS3-H55A-F	ACGCTTGCCTACGTTGCCGACTTCGGGAAAGCG
CAS3-H55A-R	CGCTTTCCCGAAGTCGGCAACGTAGGCAAGCGT
CAS3-D56A-F	CTTGCCTACGTTCACGCCTTCGGGAAAGCGACG
CAS3-D56A-R	CGTCGCTTTCCCGAAGGCGTGAACGTAGGCAAG
CAS3-K113A-F	TTCGTCGCGGTCGCCGCACATCACGGCCGGCTT
CAS3-K113A-R	AAGCCGGCCGTGATGTGCGGCGACCGCGACGAA
CAS3-D229A-F	TCGCTCGTGCTCGCAGCTAAGACGAGTGCAGCA
CAS3-D229A-R	TGCTGCACTCGTCTTAGCTGCGAGCACGAGCGA
CAS3-K315A-F	CCAACCGGAATGGGGGCAACACTCTCCGGGCTG
CAS3-K315A-R	CAGCCCGGAGAGTGTTGCCCCCATTCCGGTTGG
CAS3-D439A-F	AGTGTAGTTATCCTTGCTGAGCCACAGAGCTTG
CAS3-D439A-R	CAAGCTCTGTGGCTCAGCAAGGATAACTACACT
CAS3-E440A-F	GTAGTTATCCTTGATGCGCCACAGAGCTTGCCG
CAS3-E440A-R	CGGCAAGCTCTGTGGCGCATCAAGGATAACTAC
**For construction of CRISPR variants**	
DSP-UF	CGCGGATCCCTCGCAGACCGGGTACTAT
DSP-DR	ATAGGGTACCGGGCTTGCCGACTGAA
DSP26-UR	**AGTAGCGTTGTTGCCATTATAGACGCGATTCTCGAC**
DSP26-DF	**GTCGAGAATCGCGTCTATAATGGCAACAACGCTACT**
DSP713-UR	**TCTATCGACCAGCGCGAAGTTCATGCTCCCGCCGAG**
DSP713-DF	**CTCGGCGGGAGCATGAACTTCGCGCTGGTCGATAGA**
DSP212-UR	**TCGACCAGTCCGTAACGTATAGACGCGATTCTCGAC**
DSP212-DF	**GTCGAGAATCGCGTCTATACGTTACGGACTGGTCGA**
DSP213-UR	**TCTATCGACCAGCGCGAAATAGACGCGATTCTCGAC**
DSP213-DF	**GTCGAGAATCGCGTCTATTTCGCGCTGGTCGATAGA**
DSP114-UR	**CAAAAGTTCGGCTGAAACAACGTTATCGGAGGCGATGA**
DSP114-DF	**TCATCGCCTCCGATAACGTTGTTTCAGCCGAACTTTTG**
DSP13-UR	**AAGTTCGGCTGAAACTCGACCAGTCCGTAACGT**
DSP13-DF	**TTACGGACTGGTCGAGTTTCAGCCGAACTTTTGAAA**
**For construction of target plasmids**	
VS-F	CGCGGATCCTTCGGTCTGTGTCTCTAC
VS-R	ATAGGGTACCCACGCTCACCCATCTCACT
pCTC1-PLUS-F	GATCCCTCTTGCTAACTGCCATCAGTCATCGCCTCCGATAACGTTGGTAC
pCTC1-PLUS-R	CAACGTTATCGGAGGCGATGACTGATGGCAGTTAGCAAGAGG

pCTC13-PLUS-F	GATCCCTCTCGGCCACCACAGTCTTACTCCGCGCTGGACAGTTCCGGTAC
pCTC13-PLUS-R	CGGAACTGTCCAGCGCGGAGTAAGACTGTGGTGGCCGAGAGG
pCTC13-MINUS-F	GATCCGGAACTGTCCAGCGCGGAGTAAGACTGTGGTGGCCGAGAGGGTAC
pCTC13-MINUS-R	CCTCTCGGCCACCACAGTCTTACTCCGCGCTGGACAGTTCCG
pTCC13-PLUS-F	GATCCTCGGCCACCACAGTCTTACTCCGCGCTGGACAGTTCCGGTAC
pTCC13-PLUS-R	CGGAACTGTCCAGCGCGGAGTAAGACTGTGGTGGCCGAG
pACC13-MINUS-F	GATCCGGAACTGTCCAGCGCGGAGTAAGACTGTGGTGGCCGAGGTAC
pACC13-MINUS-R	CTCGGCCACCACAGTCTTACTCCGCGCTGGACAGTTCCG

[a] Forward and reverse primers are indicated with letters F and R, respectively. UF/UR and DF/DR are primers respectively for the upstream and downstream fragments (which were subsequently linked by a bridge PCR reaction).

[b] Restriction endonuclease cut sites are underlined. The sequences in bold within UR and DF primers are complementary to each other.

Table S3. Spacers acquired during HHPV-2 infection assay

ID[a]	Spacer sequences	Positions[b]	Strand[c]	PAM[d]
v1.1	TCACTTAGTAGCGGAGCATACGCTGACTACTCCGA	3807-3841	+	TTC
v1.2	TGATGATCGGCCCGATGCCTGAGCCGTCCGGGTCCG	8086-8121	+	TTC
v2.1	CTGAATTACAGTGAGCGTCGTAAGATGTTCGCTG	2471-2504	+	TTCG
v2.2	ACTGTACTCAAACGCTATATACAGCGGATCATCCC	4259-4225	-	TTC
v3.1	GAAGAGGGCACGCTGCCTGATCACGGTGAGTCGCTG	5941-5976	+	TTC
v3.2	GGATCGTGGAATGAAGATGAGTCGTTCCCTGAAGATC	7017-7053	+	TTC
v3.3	GTCGAGAATGCGATTGCTGATGCTGATTCTGGTCCG	6378-6413	+	TTC
v3.4	GTCTGCTGCGGTGGTGATCGGCGTGGATACCGTCG	7237-7271	+	TTC
v4.1	TCGAGCAGGACTGTCAGTCGCAGGCAACCAAGC	1935-1903	-	TTC
v4.2	CTCAATCACCTCCTCACCGGCATCCGGGTTTATCC	2395-2361	-	TTC
v5.1	GGGAGCTGTCGGTAACTGGTCCTAACGGTAATCCG	5648-5682	+	TTC
v5.2	CACGACCACGGCGCAGAATCATCAGGATCATACGCC	7132-7097	-	TTC
v5.3	AGAAGTTCGTGCTGATCGATGAGGGCAGCACGCAG	6796-6830	+	TTC
v6.1	GCATTCCCTTCGGCAATAGCTTCAGACGCAACGT	6502-6469	-	TTC
v6.2	CGTCTGATGACTTTCGGGAGCTGTCGGTAACTGGTC	5633-5668	+	TTC
v7.1	GCCTCGTCTGAAACTCGGTCGTTTGACCGGTACGA	2833-2867	+	TTC
v7.2	GACTTGTCCGGTCTGTCGCTCGGTGGCATACCCGG	4536-4570	+	TTC
v8.1	TCATCCATCACGTAGCGCAGCGAACGACCAGCACCA	737-702	-	TTC
v9.1	GCAGACCACGACGGGGGCCGGGTATAGCGAACGG	1694-1661	-	TTC
v9.2	AGTCGTGTACTGCTCACCGAGGATCAGCAGCGGAGT	2931-2896	-	TTC
v10.1	TTTCGCAGACCACGACGGGGGCCGGGTATAGCGA	1698-1665	-	TTC
v11.1	AAGCATAAAGGCAATGACCGCGTTTCTGAAGTGCGT	7748-7783	+	TTC
v12.1	CGAACTTCAGACCCGAGCGCAGTATCAGCGAACGTC	6451-6416	-	TT(C)
v12.2	GTACGTGCTGCCGTCTTGCGACGTGTTGAAAATAA	3746-3712	-	TTC
v12.3	TCAGTCGCATCAGTCCCATCAACCAGAGACAGAG	3703-3670	-	TTC
v13.1	GGAAAACGTTAGATACTGACGTTGCGTCTGAAGCTA	6451-6486	+	TTC

v13.2	TCCTGATACGCAGCCATCCAACTGTTATCAAGCGGA	464-429	-	TTA
v14.1	CTCAAGGTTGGCAAGAACATGCAGTTAGAAGAGATG	5719-5754	+	TTC
v14.2	AGCCGCGCTCGCTCTTTATCCTGATACGCAGCC	482-450	-	TTC
v14.3	AGGTGACGAATCAGTACCGCCACCATCAGCAGC	1446-1414	-	TTC
v15.1	GCTGATCGGTGGTCCGTGGTCGTCTGGTGACGCTACA	5191-5227	+	TTC
v15.2	GCTGTCTTTCGTCGAGAATGCGATTGCTGATGCTG	6368-6402	+	TTC
v16.1	GCTGATCGGTGGTCCGTGGTCGTCTGGTGACGCTA	5191-5225	+	TTC
v16.2	TCTGCTGGCTGCGTTGATCGTCGTGTTACCTCTGCGC	6720-6756	+	TTC
v17.1	CCCACTGGGAACCGCAGGCAGTACACACGACA	1366-1335	-	TTC
v17.2	TGATGATCGGCCCGATGCCTGAGCCGTCCGGGTCCG	8086-8121	+	TTC
v18.1	GGCAGCGGGTGGATAAACCCGGATGCCGGTGAGG	2351-2384	+	TTCC
v18.2	CGACTGAGGATATTGTTGATCCGATCACGGCGGCGA	3976-4011	+	TTC
v19.1	AGGATGAGCTGGGCGAGCTGCAAGATGATGAGTT	6017-6050	+	TTC
v19.2	AATACATCGGTCCTGCGCTGCTGATCGTTGGCTGC	6171-6205	+	TTC
v20.1	CAGTCAGACGCCGGTTTGTCGAGATCATGTGCATCA	989-954	-	TTC
v20.2	CGCGATCTCCCGAGCGTGATCAACTGGGGGCCGA	672-639	-	TTC
v21.1	ATCGACGTAGCACCCTCTTGGATTGCAGACTTAAG	3340-3306	-	TTC
v21.2	GGATAGTTGAGCACGTTGGTAATGATGTGTCTGCCT	7982-8017	+	TTC
v22.1	GTCTTGCGGTGATTGCTGCTGAGTCTGACTGCGGT	8137-8171	+	TTC
v22.2	ATTACAACAGTCGCGCCGCAGCGTGCGACCGTGC	234-201	-	TTC
v23.1	GAATCATTAGCGAGATATGCAGCAGCATACGAAGCA	1088-1053	-	TTC
v23.2	GCTATATGGCTGGCCGTACGGTTGATCCGGAGCGG	5286-5320	+	TTC
v24.1	CTGATCGGTTGCTCGTTGAATCTGGCGAGCACCA	1976-2009	+	TTC
v24.2	GCTATATGGCTGGCCGTACGGTTGATCCGGAGCGGG	5286-5321	+	TTC
v25.1	CTCAAGGTTGGCAAGAACATGCAGTTAGAAGAGAT	5719-5753	+	TTC
v26.1	GAAAAACGTTAGATACTGACGTTGCGTCTGAAGCT	6451-6485	+	TTC
v27.1	GCGCTCTTGTTCAGGTGACGAATCAGTACCGCCAC	1458-1424	-	TTC
v28.1	TGTCCGTTCCTGTTGGTGAGGGTTCCCACCCACCCA	7352-7384	+	TTC
v28.2	CGTCGCGCAAAGCGGACAGGTATCAGGCTCAGCGTG	1575-1540	-	TTC
v29.1	ACTCCGTTAGCAAAACGCTTTGCGAAACTGAACGTG	6873-6908	+	TTC
v30.1	CGGTATGTGGCTCACCTTGAGTCCACCGGACCCACT	796-761	-	TTC
v31.1	CAGCCGCGCTCGCTCTTTATCCTGATACGCAGCCAT	483-448	-	TT(C)
v31.2	AGAAACGCGGTCATTGCCTTTATGCTTGAACGCCGCAA	7774-7737	-	TTC
v32.1	GCAGACCACGACGGGGGCCGGGTATAGCGAACGG	1694-1661	-	TTC
v33.1	CAGTCAGACGCCGGTTTGTCGAGATCATGTGCAT	989-956	-	TTC
v33.2	AGCATCAGGTGTCTCAGTTACCTCAGGTGCATCAGTT	4849-4813	-	TTC
v34.1	ATACACAAGCACAGGACACCTCCCGCGGACCCACAG	7458-7423	-	TTC
v34.2	GCTGATACTGCGCTCGGGTCTGAAGTTCGGAAAACG	6423-6458	+	TTC
v35.1	GCCAAACACAGCTCGAGCTGCAAGAAGAGTCTCAATC	4483-4519	+	TTC
v35.2	GTCTGCTGCGGTGGTGATCGGCGTGGATACCGTCGC	7237-7272	+	TTC
v36.1	ACTAAGCGAGTGACGTTGACTGATGCTGGTGCGTT	5000-5034	+	TTC
v36.2	AATACATCGGTCCTGCGCTGCTGATCGTTGGCTG	6171-6204	+	TTC
v37.1	GACTTGTCCGGTCTGTCGCTCGGTGGCATACCCGG	4536-4570	+	TTC

v37.2	GCTATATGGCTGGCCGTACGGTTGATCCGGAGCGG	5286-5320	+	TTC
v37.3	AAGCATAAAGGCAATGACCGCGTTTCTGAAGTTCGT	7748-7783	+	TTC
v38.1	CGTCGCGCAAAGCGGACAGGTATCAGGCTCAGCGT	1575-1541	-	TTC
v39.1	AATACATCGGTCCTGCGCTGCTGATCGTTGGCTGCGT	6171-6207	+	TTC
v40.1	AGCCGCGCTCGCTCTTTATCCTGATACGCAGCCATCC	482-446	-	TTC
v40.2	CGCGATCTCCCGAGCGTGATCAACTGGGGGCCGA	672-639	-	TTC
v41.1	GATTGTTGCTGTCGTCTTGATCGGGTCCGGTCTGA	5412-5446	+	TTC
v42.1	GTCTGCTGCGGTGGTGATCGGCGTGGATACCGTCG	7237-7271	+	TTC
v43.1	GGTAAATAGAGGTATCCAAAATGCAATTTTTTAAAAT	4617-4653	+	TTC
v43.2	AATACATCGGTCCTGCGCTGCTGATCGTTGGCTG	6171-6204	+	TTC
v44.1	GAATCATTAGCGAGATATGCAGCAGCATACGAAG	1088-1055	-	TTC
V45.1	CACGATCCGAAGAACTCAGCAACCTCAGGCTCGCTC	7024-6989	-	TTC
V46.1	GCACGGTGATCGAGAAACACGTAGAGCTGTGTGATG	908-943	+	TTC
V46.2	GGTAAATAGAGGTATCCAAAATGCAATTTTTTAAAA	4617-4652	+	TTC
V46.3	GCTGATACTGCGCTCGGGTCTGAAGTTCGGAAAACG	6423-6458	+	TTC
V47.1	ATACAGTTTTTTGGAGAACACATTTACGCGCTCTT	324-290	-	TTC
V48.1	AGTGGTGAGTTGACTGCGCTCGAGGAAACACAGT	7062-7095	+	TTC
V48.2	TTGATGCGGTTTCACTACTGTCCGATGCGCGAGCGG	7481-7516	+	TTC
V49.1	ATCGAGATCCGCAGCGAACTCATCATCTTGCAACTC	6066-6035	-	TTC
V49.2	AAGCGATTGGCTACGTTGCCTTTCTTTAAGAGCGA	6960-6994	+	TTC
V50.1	GTGGGACGCTTGATCCTGATGCTGATGAACAGCGT	7790-7816	+	TTC
V51.1	GTCGAGAATGCGATTGCTGATGCTGATTCTGGTCCG	6378-6413	+	TTC
V51.2	CGGTATGTGGCTCACCTTGAGTCCACCGGACCCACTG	796-760	-	TTC
V52.1	CTTTTTGCGTATTGGACGCCCACATGGTAGCCGC	1162-1129	-	TTC
V53.1	ACTATCGAAACACAGGCGTTTACGATTATTCGGTCTG	5866-5902	+	TTC
V54.1	CGCTTGATAACAGTTGGATGGCTGCGTATCAGGA	431-464	+	TTC
V55.1	AAGAGGGCACGCTGCCTGATCACGGTGAGTCGCT	5942-5975	+	TTCG
V56.1	GGTAAATAGAGGTATCCAAAATGCAATTTTTTAAA	4617-4651	+	TTC

[a] Each spacer is designated with a 'v' (indicating virus-derived) followed by two numbers (separated by a dot). The first is the colony number, and the second indicates its location within the expanded array according to the leader (e.g. "v1.1" is the most recently acquired spacer in colony1).

[b] The positions of protospacers (from which a spacer was derived) on the HHPV-2 genome.

[c] "+" and "-" correspond respectively to the coding strand and the template strand of the *rep* gene (ORF1).

[d] The C in parenthesis is the first nucleotide of the following protospacer.

Table S4. Spacers acquired during pVS (a plasmid carrying a viral sequence) transformation assay

ID[a]	Spacer sequences	Positions[b]	Strand[c]	PAM

p1.1	AGTCAGTACGATAGGACCACGGTCTCGCGGCGATT	4810-4844	+	TTC
p2.1	CGTGTGCGTAGGCGATGGTCTCGCGGAGCGAGCGC	7703-7669	-	TTC
p3.1	TCCCATCGAGTGTTACGAATCCGTGCAACGACACCC	4646-4681	+	TTC
p4.1	CGCGCACATTTCCCCGAAAAGTGCCACCTGACGT	2988-3021	+	TTC
p5.1	CCCGAAAAGTGCCACCTGACGTCTAAGAAACCA	3000-3032	+	TTC
p5.2	GACCAGGTCCTTACCGAGGACCGTCTCGCGGAACTTG	5356-5392	+	TTC
p6.1	AGGTCGAGGTGGCCCGGCTCCATGCACCGCGACGC	710-676	-	TTC
p7.1	TCTGGGTTTGCACGGACACCGTACGACGTAGCCC	6091-6124	+	TTC
p8.1	ACGAGGACGCGCCGGAGATTGCGCTCAAAGTCCCTG	5957-5992	+	TTC
p9.1	GTGCGTCGCGTCGATGATGCGGCGGTTGAACGCCC	7612-7578	-	TTC
p10.1	ACGAGGACGCGCCGGAGATTGCGCTCAAAGTCCCTG	5957-5992	+	TTC
p11.1	AAGAACTCTGTAGCACCGCCTACATACCTCGCTC	1674-1641	-	TTC
p12.1	TTCTCGCTTCCGGCGGCATCGGGATGCCCGCGTTG	447-481	+	TTC
p12.2	TCCCATCGAGTGTTACGAATCCGTGCAACGACACC	4646-4680	+	TTC
p13.1	ACTCGAGACGTCCTCTTTGAGACGCTAGAAGCGC	6475-6508	+	TTC
p14.1	CGTGTGCGTAGGCGATGGTCTCGCGGAGCGAGCGCCA	7703-7667	-	TTC
p15.1	TTGAAGTGGTGGCCTAACTACGGCTACACTAGAAG	1673-1707	+	TTC
p15.2	CAAGGCGCGGGAAGGCCGACGGTCATACGATGAG	3782-3815	+	TTCT
p16.1	AGACGCTGTCTATTGGGCCGGAGATTGCACAGCAA	6662-6696	+	TTC
p17.1	TCAAGGCGCGGGAAGGCCGACGGTCATACGATGAGA	3781-3816	+	TTC
p17.2	CACATGAGGACTACGAGTGTGAGACGTCCGAGTAC	5534-5568	+	TTG
p18.1	AACCGCCGCATCATCGACGCGACGCACGAACACGCG	7586-7621	+	TTC
p19.1	GACGAGACAGAGAGTCAATTTGCGCTTCTCGAGG	4204-4237	+	TTC
p19.2	GGGAATCGGGCGGACCACGTCGCCGGCGTAATA	5893-5925	+	TTC
p20.1	AGCGTCTCAAAGAGGACGTCTCGAGTGAACGACT	6500-6467	-	TTCT
p20.2	TCGAGGCACTACAAACAGCGGCGGACGGTGTCGACA	4232-4267	+	TTC
p21.1	GTGCGTCGCGTCGATGATGCGGCGGTTGAACGC	7612-7580	-	TTC
p22.1	TCCGCAAGAATTGATTGGCTCCAATTCTTGGAGTG	781-747	-	TTC
p22.2	CGAATACCGCAAGCGACAGGCCGATCATCGTCGCG	287-253	-	TTC
p23.1	GTGCACACAGCCCAGCTTGGAGCGAACGACCTAC	1525-1492	-	TTC
p23.2	TCTGGGTTTGCACGGACACCGTACGACGTAGCCCA	6091-6125	+	TTC
p24.1	TGAGAATAGTGTATGCGGCGACCGAGTTGCTCTTGCC	2597-2633	+	TTC
p25.1	ATCGAGTAATCTTCGACGAGACAGAGAGTCAATTT	4190-4224	+	TTC
p26.1	TCAAGGCGCGGGAAGGCCGACGGTCATACGATGAG	3781-3815	+	TTC
p26.2	TCCCATCGAGTGTTACGAATCCGTGCAACGACACCC	4646-4681	+	TTC
p27.1	ACTCGAGACGTCCTCTTTGAGACGCTAGAAGCGC	6475-6508	+	TTC
p28.1	GTGGGTCTCGAAACCTCGGGTGGTTGGCTGACTCT	5423-5457	+	TTC
p29.1	TCCCATCGAGTGTTACGAATCCGTGCAACGACACC	4646-4680	+	TTC
p30.1	GGGAATCGGGCGGACCACGTCGCCGGCGTAATAGA	5893-5927	+	TTC
p31.1	CGGGTCTGTACCTGTCGAATCGGTCCCTGACGAAAG	7245-7280	+	TTC
p32.1	GGATAGTTGAGCACGTTGGTAATGATGTGTCTGCC	6979-7013	+	TTC
p32.2	ATTTTTAATTTAAAAGGATCTAGGTGAAGATCCTT	1976-1942	-	TTC
p33.1	GCCCGACACATTGCCCGTGTTACTCGTGCAACCG	6050-6017	-	TTC

p34.1	GTACGCAGAGACGGTCTCAGCGGCGAAGACACCGC	5148-5182	+	TTC
p35.1	ATTTTTAATTTAAAAGGATCTAGGTGAAGATCCTT	1976-1942	-	TTC
p36.1	ATGCTGACCGAGTCGTTCACTCGAGACGTCCTCTT	6457-6491	+	TTC
p37.1	CGAAGGTAACTGGCTTCAGCAGAGCGCAGATACCAA	1751-1716	-	TTC
p37.2	CTGTTTTTGCTCACCCAGAAACGCTGGTGAAAGTAA	2828-2793	-	TTC
p37.3	AATCGCCGCGAGACCGTGGTCCTATCGTACTGACTGA	4844-4808	-	TTC
p38.1	AAGAACTCTGTAGCACCGCCTACATACCTCGCTCTG	1674-1639	-	TTC
p39.1	ATGATTAGAGTAGGGTCGACGAACTCTGAACCTATG	6808-6843	+	TTC
p40.1	CGTGTGCGTAGGCGATGGTCTCGCGGAGCGAGCGC	7703-7669	-	TTC
p40.2	TGCTAACCAGTAAGGCAACCCCGCCAGCCTAGCCG	956-922	-	TTC
p41.1	CGCGAGACGGTCCTCGGTAAGGACCTGGTCGAACG	5385-5351	-	TTC
p42.1	ACTCGAGACGTCCTCTTTGAGACGCTAGAAGCGCTT	6475-6510	+	TTC
p42.2	CTGCGTTATCCCCTGATTCTGTGGATAACCGTATT	1199-1165	-	TTC
p43.1	ACTCGAGACGTCCTCTTTGAGACGCTAGAAGCGCTT	6475-6510	+	TTC
p43.2	TCCCATCGAGTGTTACGAATCCGTGCAACGACACC	4646-4680	+	TTC
p44.1	AGACGGATCAGACCCGAGACAGCAGACCGGACA	7046-7014	-	TTC
p45.1	GGCTTGTAGGCCGCCGCGTGTTCGTGCGTCGCGTCG	7635-7600	-	TTC
p46.1	CCGCCTGTCCCGCAGCCTTGTCGAAGTGTTCTCCC	8216-8182	-	TTCG
p47.1	TCGACAAAGGAGCGTTGTACCTCGAAGAATTAGCC	4916-4950	+	TTC
p48.1	GGTGTAGGTCGTTCGCTCCAAGCTGGGCTGTGTGCA	1489-1524	+	TTC
p49.1	ACTCGCGAGTCTCCCGTCGAAGCGTCTCTTAGAGG	6195-6229	+	TTC
p50.1	CTCGCCGGTTAGGTGGGAGAGTTCGCCCGATTCGA	3151-3185	+	TTC
p51.1	AGACGCTAGAAGCGCTTGATGAGTTGACCGAGGAGG	6494-6529	+	TTG
p52.1	ACTCGTCTATTGGGCTATAGCAACCCTCAACAGACG	4453-4488	+	TTC
p52.2	GGCTTGTAGGCCGCCGCGTGTTCGTGCGTCGCGT	7635-7602	-	TTC
p53.1	AGGTCGAGGTGGCCCGGCTCCATGCACCGCGACGC	710-676	-	TTC
p54.1	GGTCGAGGAGGTCGAGGTCGAGGTTAATGTAAAACG	3292-3257	-	TTC

[a] Each spacer is designated with a ‘p’ (indicating plasmid-derived) followed by two numbers (separated by a dot). The first is the colony number, and the second indicates its location according to the leader (e.g. “p1.1” is the most recently acquired spacer in colony1).

[b] The positions of protospacers (from which a spacer was derived) on the target plasmid pVS. The fourth nucleotide of the unique SphI restriction site was taken as the first position. The priming spacer13 imperfectly matches to the nucleotides 7209-7245.

[c] “+” and “-” correspond respectively to the coding strand and the template strand of the *pyrF* gene.

Table S5. Distribution of the theoretical PAM (TTC) and the observed protospacers on the HHPV-2 genome or the target plasmid pVS.

Target	Region[a]	Positions	PAM	PAM	Protospacer	Protospacer

			(+)[b]	(-)[b]	(+)[b]	(-)[b]
**HHPV-2**	**non-target strand preferred**	4401-35	73	65	50	8
	**target strand preferred**	73-2278	27	35	3	25
	**non-preferred**	2279-4400	33	38	5	6
**pVS**	**non-target strand preferred**	4600-7208	36	55	34	5
	**target strand preferred**	7209-2000	56	55	5	20
	**non-preferred**	2001-4659	54	34	4	2

[a] The HHPV-2 genome or pVS was artificially divided into three regions (depicted in Figure 6 of the main text) according to the strand bias during spacer acquisition. The non-target strand and target strand of the priming protospacer respectively correspond to the coding strand and the template strand of the *rep* gene (HHPV-2) or the *pyrF* gene (pVS).

[b] (+) or (-) indicates the location on the coding or template strand of the *rep* gene (HHPV-2) or the *pyrF* gene (pVS).

Table S6. Imperfect matches between haloarchaeal spacers and haloviral genomes

Strains	CRSIRP_ID[a]	Spacer_ID	Spacer length	Spacer st-end	Target virus	Target seq st-end	E value
Halorubrum lacusprofundi ATCC 49239	NC_012030_1	spacer16	35	1-16	BJ1	27701-27686	0.002
		spacer31	35	17-31	BJ1	13458-13444	0.007
		spacer55	34	13-27	SH1	4113-4127	0.007
		spacer58	36	1-16	PhiCH1	35796-35811	0.002
		spacer61	36	9-24	PhiCH1	19112-19127	0.002
		spacer65	37	16-30	BJ1	33129-33115	0.007
		spacer120	34	18-32	BJ1	8774-8788	0.007
	NC_012030_4	spacer6	33	5-24	PhiCH1	31314-31295	0.002
		spacer32	33	5-24	PhiCH1	31314-31295	0.002
		spacer68	36	17-31	SH1	27245-27259	0.007
		spacer72	36	17-31	SH1	27245-27259	0.007
		spacer78	35	6-26	BJ1	39722-39702	4.00E-04
Haloarcula marismortui ATCC 43049	NC_006391_2	spacer2	36	6-21	BJ1	31264-31249	0.002
		spacer2	36	7-21	HRPV-2	2418-2404	0.007
		spacer2	36	7-21	HRPV-2	9382-9368	0.007
		spacer2	36	7-21	HRPV-2	9675-9661	0.007
		spacer14	36	2-16	BJ1	29267-29281	0.007
		spacer20	36	15-30	HRPV-2	3061-3076	0.002
	NC_006392_2	spacer7	37	21-35	SH1	17729-17743	0.007
	NC_006392_3	spacer19	36	2-17	SH1	22417-22402	0.002
		spacer19	36	2-16	SH1	3814-3828	0.007
		spacer19	36	3-17	SH1	10751-10737	0.007
		spacer21	35	13-27	BJ1	38090-38076	0.007
Haloferax	NC_017941_4	spacer16	38	7-21	HF1	50431-50417	0.008

mediterranei ATCC 33500	NC_017944_2	spacer3	35	20-34	BJ1	36527-36513	0.007
		spacer17	36	14-28	PhiCH1	3099-3085	0.007
		spacer19	34	11-26	PhiCH1	57678-57663	0.002
		spacer19	34	11-25	BJ1	5143-5129	0.007
	NC_017941_5	spacer4	36	11-25	SH1	23972-23986	0.007
		spacer15	37	6-20	SH1	993-1007	0.007
		spacer18	36	17-32	SH1	5983-5968	0.002
	NC_017941_7	spacer22	36	18-32	SH1	23431-23417	0.007
	NC_017944_1	spacer3	36	13-27	PhiCH1	3207-3193	0.007
Halomicrobium mukohataei DSM 12286	NC_013202_12	spacer2	35	3-28	BJ1	28797-28822	5.00E-07
		spacer4	36	20-34	BJ1	5251-5237	0.007
		spacer14	34	1-15	BJ1	5326-5312	0.007
		spacer26	35	10-26	PhiCH1	40520-40536	4.00E-04
		spacer32	33	5-23	SH1	15647-15629	0.006
	NC_013201_1	spacer4	35	2-24	BJ1	27057-27080	0.002
		spacer4	35	9-23	HF2	59049-59063	0.007
		spacer4	35	9-23	HF1	57278-57292	0.007
		spacer10	36	10-24	HRPV-3	5049-5035	0.007
Halorhabdus utahensis DSM 12940	NC_013158_1	spacer21	35	7-21	BJ1	3863-3877	0.007
		spacer21	35	4-18	SH1	2544-2558	0.007
		spacer24	35	6-25	HHPV-1	7066-7047	0.002
		spacer24	35	11-30	HRPV-2	7155-7136	0.002
		spacer24	35	11-30	HRPV-6	7107-7088	0.002
		spacer28	37	2-16	HF2	13880-13894	0.007
		spacer28	37	2-16	HF1	13880-13894	0.007
		spacer28	37	23-37	PhiCH1	40500-40486	0.007
		spacer35	37	1-28	BJ1	29670-29697	8.00E-06
		spacer35	37	2-18	BJ1	1359-1343	5.00E-04
		spacer35	37	1-15	BJ1	4992-5006	0.007
		spacer39	35	13-31	PhiCH1	41010-41028	3.00E-05
		spacer41	37	21-35	PhiCH1	8248-8262	0.007
		spacer44	34	16-30	PhiCH1	53320-53306	0.007
Haloferax volcanii DS2	NC_013967_2	spacer10	37	13-31	SH1	12253-12235	0.007
	NC_013966_1	spacer6	36	7-21	HF2	62924-62938	0.007
		spacer7	37	11-25	HRPV-2	152-138	0.007
		spacer7	37	11-25	HRPV-6	165-151	0.007
		spacer14	35	6-20	HF2	51950-51964	0.007
		spacer35	36	7-21	BJ1	10520-10506	0.007
		spacer38	36	1-15	PhiCH1	19658-19672	0.007
Natrialba magadii ATCC 43099	NC_013922_3	spacer1	37	8-22	PhiCH1	23586-23572	0.007
		spacer3	35	14-30	BJ1	26576-26560	4.00E-04
		spacer3	35	20-35	HF1	52964-52979	0.002
		spacer3	35	7-21	PhiCH1	12711-12697	0.007

		spacer8	38	7-21	HF2	5473-5487	0.008
		spacer8	38	7-21	HF1	5473-5487	0.008
		spacer16	36	16-30	PhiCH1	13524-13510	0.007
		spacer27	36	19-33	BJ1	29256-29242	0.007
	NC_013923_2	spacer6	36	12-29	HF2	9776-9759	1.00E-04
		spacer6	36	12-29	HF1	9776-9759	1.00E-04
		spacer10	34	16-32	PhiCH1	54194-54178	4.00E-04
Haloarcula hispanica ATCC 33960	NC_015943_1	spacer13	37	9-30	HHPV-2	44-65	5.00E-04
Natronomonas pharaonis DSM 2160	NC_007427_1	spacer1	36	16-30	HF2	75526-75512	0.007
		spacer1	36	16-30	HF1	73754-73740	0.007

[a] The CRISPR designations follow the IDs used in the CRISPRdb database (http://crispr.u-psud.fr/crispr/). Orientations and spacer numbering of NC_015943_1, NC_017941_7, and NC_017944_1 arrays (from *H. hispanica* and *H. mediterranei*) have been manually calibrated.

SUPPLEMENTARY REFERENCES

1. Sinkunas, T., Gasiunas, G., Fremaux, C., Barrangou, R., Horvath, P. and Siksnys, V. (2011) Cas3 is a single-stranded DNA nuclease and ATP-dependent helicase in the CRISPR/Cas immune system. *EMBO J.*, **30**, 1335-1342.
2. Liu, H., Han, J., Liu, X., Zhou, J. and Xiang, H. (2011) Development of pyrF-based gene knockout systems for genome-wide manipulation of the archaea *Haloferax mediterranei* and *Haloarcula hispanica*. *J. Genet. Genomics*, **38**, 261-269.
3. Cai, S., Cai, L., Liu, H., Liu, X., Han, J., Zhou, J. and Xiang, H. (2012) Identification of the haloarchaeal phasin (PhaP) that functions in polyhydroxyalkanoate accumulation and granule formation in *Haloferax mediterranei*. *Appl. Environ. Microbiol.*, **78**, 1946-1952.
